# Blood Plasma, Fibrinogen or Fibrin Biomaterial for the Manufacturing of Skin Tissue-Engineered Products and Other Dermatological Treatments: A Systematic Review

**DOI:** 10.3390/jfb16030079

**Published:** 2025-02-22

**Authors:** Álvaro Sierra-Sánchez, Raquel Sanabria-de la Torre, Ana Ubago-Rodríguez, María I. Quiñones-Vico, Trinidad Montero-Vílchez, Manuel Sánchez-Díaz, Salvador Arias-Santiago

**Affiliations:** 1 Unidad de Producción Celular e Ingeniería Tisular, Virgen de las Nieves University Hospital, Andalusian Network of Design and Translation of Advanced Therapies, 18014 Granada, Spain; alvarosisan@gmail.com (Á.S.-S.); salvadorarias@ugr.es (S.A.-S.); 2 Instituto de Investigación Biosanitaria ibs.GRANADA, 18012 Granada, Spain; 3 Department of Dermatology, Virgen de las Nieves University Hospital, 18012 Granada, Spain; 4 Wake Forest Institute for Regenerative Medicine, Wake Forest School of Medicine, Medical Center Boulevard, Winston-Salem, NA 27101, USA; 5 Department of Biochemistry and Molecular Biology IIi and Immunology, University of Granada, 18071 Granada, Spain; 6 Department of Dermatology, University of Granada, 18016 Granada, Spain

**Keywords:** biomaterial, blood plasma, fibrin, fibrinogen, tissue-engineered skin substitute, scaffold

## Abstract

The use of blood plasma, fibrinogen or fibrin, a natural biomaterial, has been widely studied for the development of different skin tissue-engineered products and other dermatological treatments. This systematic review reports the preclinical and clinical studies which use it alone or combined with other biomaterials and/or cells for the treatment of several dermatological conditions. Following the PRISMA 2020 Guidelines, 147 preclinical studies have revealed that the use of this biomaterial as a wound dressing or as a monolayer (one cell type) skin substitute are the preferred strategies, mainly for the treatment of excisional or surgical wounds. Moreover, blood plasma is mainly used alone although its combination with other biomaterials such as agarose, polyethylene glycol or collagen has also been reported to increase its wound healing potential. However, most of the 17 clinical reviewed evaluated its use for the treatment of severely burned patients as a wound dressing or bilayer (two cell types) skin substitute. Although the number of preclinical studies evaluating the use of blood plasma as a dermatological treatment has increased during the last fifteen years, this has not been correlated with a wide variety of clinical studies. Its safety and wound healing potential have been proved; however, the lack of a standard model and the presence of several approaches have meant that its translation to a clinical environment is still limited. A higher number of clinical studies should be carried out in the coming years to set a standard wound healing strategy for each dermatological disease.

## 1. Introduction: Tissue Engineering and the Human Blood Plasma, Fibrinogen or Fibrin as a Biomaterial in Dermatology

Tissue engineering (TE) is an interesting and growing multidisciplinary field which involves several areas such as cell biology, material or biomaterial science, engineering, or medicine. It appears as a necessity to solve the lack of organ donors or another efficient substitute for the tissue required. At the beginning, TE was focused on the search for useful materials for engraftment but without functionality [1]. However, in recent years, the field has evolved to the practice of combining materials, cells and biologically active molecules [2].

Therefore, the design of TE strategies requires biomaterials or scaffolds with a three-dimensional (3D) structure to guide tissue formation into the desired shape but allowing the transport of nutrients and growth factors which promote tissue growth [3]. Moreover, these materials must resemble and mimic the function of the natural extracellular matrix (ECM) [4], the relation between the material and the biologic environment should be considered [5] and they must be characterized for preserving cell viability, adhesion and differentiation [6,7].

One of the main fields where biomaterials have been and are applied is the research and treatment of several dermatological pathologies. They have been used alone or combined with cells for the development of advanced therapies, referred as tissue-engineered skin substitutes (TESSs). These are defined as any safe product, constituted of human skin cells and/or biomaterials, capable of replacing damaged human skin and resembling its structural and functional characteristics [8,9]. Therefore, biomaterials used in dermatology must resemble and mimic the function of the natural ECM [4,10,11], support keratinocyte proliferation and provide the right environment for fibroblasts growth, apart from promoting recruiting/seeding, adhesion, proliferation, differentiation and neo-tissue genesis [10,11].

To that purpose, three types of biomaterials, natural, synthetic and composite (the combination of natural and synthetic), have been analyzed [11]. Among these, natural biomaterials such as collagen, hyaluronic acid or blood plasma, fibrinogen or fibrin are preferred [12] because they contain protein motifs that facilitate cell adhesion, are naturally found in human body and have demonstrated better compatibility and degradation in vivo [13].

**Figure 1:**
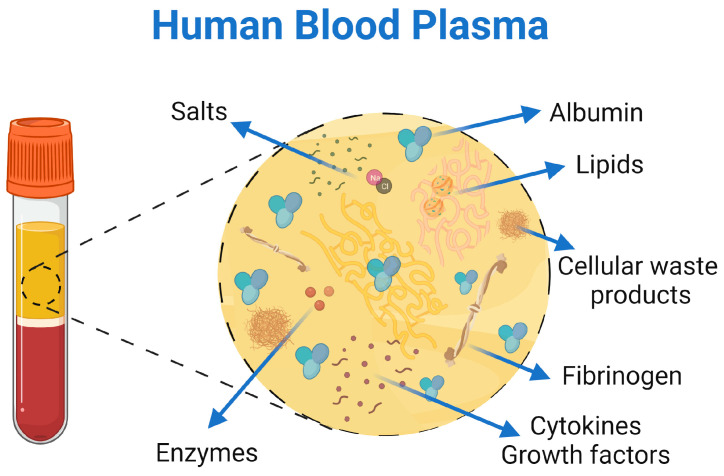
Schematic representation of the human blood plasma composition. *Created in BioRender. Sierra-Sánchez, Á. (2025) https://BioRender.com/e61b799*.

In the case of blood plasma, fibrinogen or fibrin, several animal sources of this biomaterial have been studied. However, since the purpose of the TESSs is to treat patients, human origin is the most studied and well known, avoiding the application of animal origin biomaterials and their associated ethical concerns for clinical purposes. Human blood plasma is defined as an acellular light-yellow liquid that carries the blood components throughout the body and contains water (90%), proteins, carbohydrates, lipids, salts, enzymes, nutrients or waste products from the blood cells, among others (Figure 1). Therefore, it is the most complex human-derived proteome and, for this reason, it is used for the preparation of many therapeutic products [14]. However, due to the variability between donors, its protein profile characterization has been limited [14,15], which could be a disadvantage in terms of standardization.

**Figure 2:**
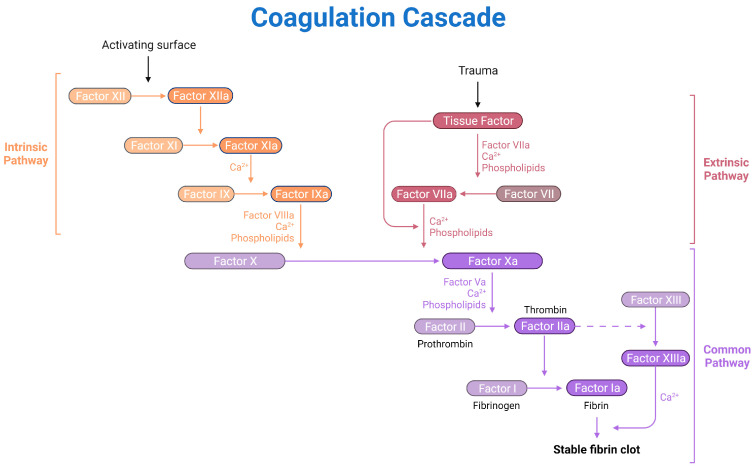
Schematic representation of the coagulation cascade physiological process. *Created in BioRender. Sierra-Sánchez, Á. (2025) https://BioRender.com/l20w744*.

The usefulness of human plasma as a biomaterial for dermatological purposes lies in the fact that it is an acellular component, so immune rejection is avoided [16]. Moreover, the presence of the clotting proteins such as fibrinogen and thrombin, enables generating a provisional hydrogel that closely mimics ECM [17], due to a homeostasis process referred as a “coagulation cascade” (Figure 2). Interestingly, this can be triggered in vitro by several procedures such as crosslinking via enzymes or UV radiation [18,19] or adding Ca^2+^ [20,21], allowing the generation of a dermal matrix that can be used for TESSs or hydrogel manufacture. Therefore, the application of this natural biomaterial over synthetics is preferred due to its controllable degradation rate, nontoxic degradation products, and excellent biocompatibility. Moreover, the morphology, mechanical properties, and stability of these hydrogels could be tuned, and, regarding the biological properties, they present high cell seeding efficiency, uniform cell distribution, adhesive properties and improved cellular interaction [22].

Regarding the blood plasma-based hydrogel formation, fibrinogen, a large, complex, fibrous glycoprotein with three pairs of polypeptide chains (Aα, Bβ and γ), the enzyme thrombin, which cleaves the small peptides A and B from α and β chains, respectively, and the fibrin monomer, as the yielded product of this enzymatic reaction [23,24] are the key factors required for generating the 3D clot filamentous network [25].

The biological characteristics of this temporary hydrogel, apart from being a scaffold, allow the binding of several proteins and growth factors located into and around the wounds, useful in the healing process. Among them, fibronectin, fibroblast growth factor (FGF) and vascular endothelial growth factor (VEGF) enable this matrix to play an active role in wound healing through specific interactions with cells [26]. Moreover, the addition of natural components to this ECM, such as collagen, fibronectin, hyaluronic acid or keratins can impart cell instructions cues not found in a fibrin gel alone [27].

Regarding the mechanical properties of a fibrin matrix, in a physiological environment, fibrin clots must stop bleeding but allow cell penetration, which means that they should have a viscoelastic behavior [23,26]. However, it is difficult to determine standard mechanical values, since each fibrin matrix depends on the nature of human blood plasma and the composition of each donor, which means a high inter-variability. Despite this, it is well known how different forces, strains and the modulation of the concentration of the different components of the human plasma affect the fibrin matrix structure and properties [23,26,27], which could be useful to generate hydrogels with different properties depending on the purpose. Moreover, the incorporation of cells such as fibroblasts into them also influences their stiffness, resulting in a small increase [28], indicating that the manufacture of more complex plasma-based approaches could be a better strategy for dermatological clinical treatments.

Hydrogel degradation is also an important fact, since the fibrin is highly susceptible to proteolytic cleavage mediated by plasmin and matrix metalloproteinases naturally present in the human plasma [27]. Therefore, this process must be controlled to achieve the desired effect for tissue regeneration. Several inhibitors such as tranexamic acid [29], aprotinin and pharmacological metalloproteinases inhibitors [27] have been included to slow proteolysis. However, for dermatological purposes, the degradation of fibrin is useful because it releases products which have been shown to stimulate vascular smooth muscle cell proliferation as well as collagen and elastin deposition [30], important for skin wound healing.

Therefore, blood plasma, fibrinogen or fibrin is a useful natural polymer for skin wound healing and its use has been widely studied for several dermatological purposes since a long time ago. The aim of this systematic review is to provide a whole vision of how this biomaterial has been and is being investigated in the field of dermatology for the development of basic, translational and clinical approaches.

## 2. Materials and Methods

**Figure 3:**
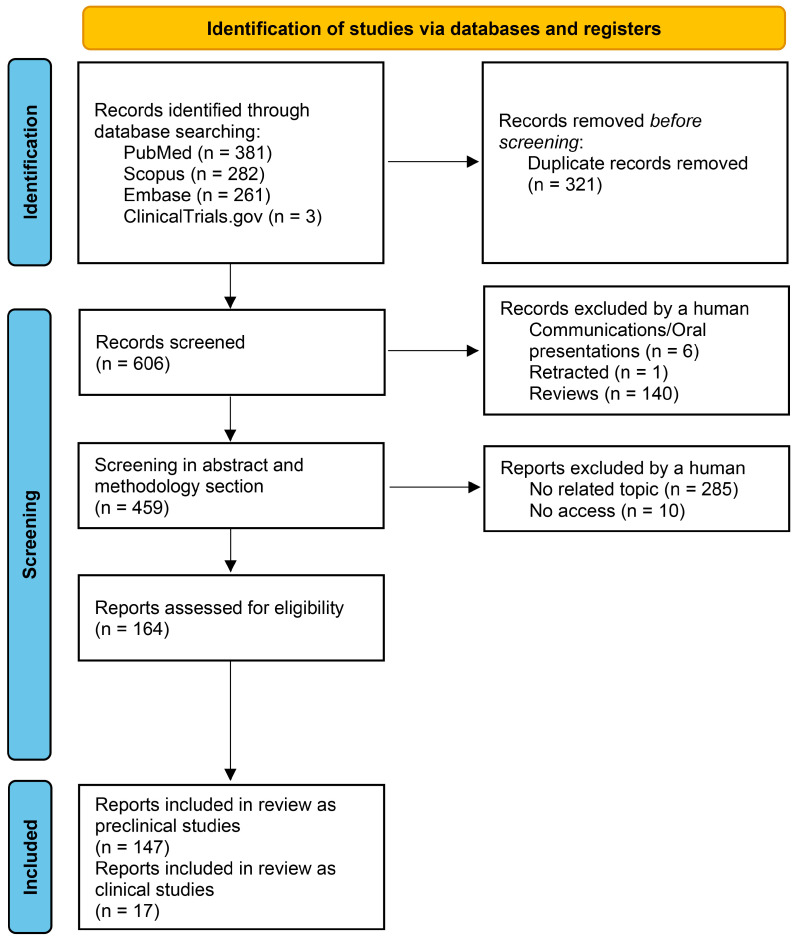
PRISMA workflow applied to this study.

To perform the systematic literature review, the PRISMA 2020 guidelines were followed (Figure 3). Four databases, PubMed (Medline), Scopus, Embase and ClinicalTrials.gov, were searched for preclinical and clinical studies where blood plasma, fibrinogen or fibrin was studied as a biomaterial for the development of TESSs and other dermatological treatment approaches, using the following search strings (adapted for each database) without time limits:

PubMed (Medline): ((skin AND substitute OR (artificial AND skin) OR (tissue AND engineered AND skin AND substitute) OR (tissue AND engineered AND skin) OR (tissue AND engineering)) AND human AND plasma OR fibrin) AND (hydrogel OR biomaterial) AND skin. Filters: English, Spanish.

Scopus: ((skin AND substitute OR (artificial AND skin) OR (tissue AND engineered AND skin AND substitute) OR (tissue AND engineered AND skin) OR (tissue AND engineering)) AND human AND plasma OR fibrin) AND (hydrogel OR biomaterial) AND skin. Filters: English, Spanish.

Embase: ((skin AND substitute OR (artificial AND skin) OR (tissue AND engineered AND skin AND substitute) OR (tissue AND engineered AND skin) OR (tissue AND engineering)) AND human AND plasma OR fibrin) AND (hydrogel OR biomaterial) AND skin AND [english]/lim AND ([article]/lim OR [article in press]/lim OR [data papers]/lim). Filters: English, Spanish.

ClinicalTrials.gov: ((skin AND substitute OR (artificial AND skin) OR (tissue AND engineered AND skin AND substitute) OR (tissue AND engineered AND skin) OR (tissue AND engineering)) AND human AND plasma OR fibrin) AND (hydrogel OR biomaterial) AND skin. Filters: English, Spanish.

The systematic review was performed in August 2024 (latest search 4 August 2024). The indexing databases were filtered, and the duplicated studies were removed. Then, the communications, oral presentations, retracted studies and reviews were removed. Those studies that could not be accessed were also excluded and the remaining were classified according to their abstracts into preclinical and clinical studies. Preclinical studies were defined as those where in vitro (without including animal testing) or in vivo (animal experimentation) analysis were performed including the blood plasma, fibrinogen or fibrin as a biomaterial for dermatological purposes. These included aspects such as mechanical characterization, cell viability or cell metabolic activity. On the other hand, clinical studies were considered as those which included or recruited patients for the evaluation of this biomaterial, with or without cells, as a dermatological treatment. The list of studies was independently reviewed and classified by at least three of the authors to reduce the risk of bias. No related topic studies were also discarded (Figure 3). All these steps were performed based on the bibliographic search protocols developed by Page et al. [31] and the PRISMA 2020 guidelines.

The detailed protocol was registered at Open Science Framework (https://osf.io/) and the information can be found at https://doi.org/10.17605/OSF.IO/W7QSD.

## 3. Results

### 3.1. Preclinical Use of the Blood Plasma, Fibrinogen or Fibrin for Dermatological Treatment Approaches

**Figure 4:**
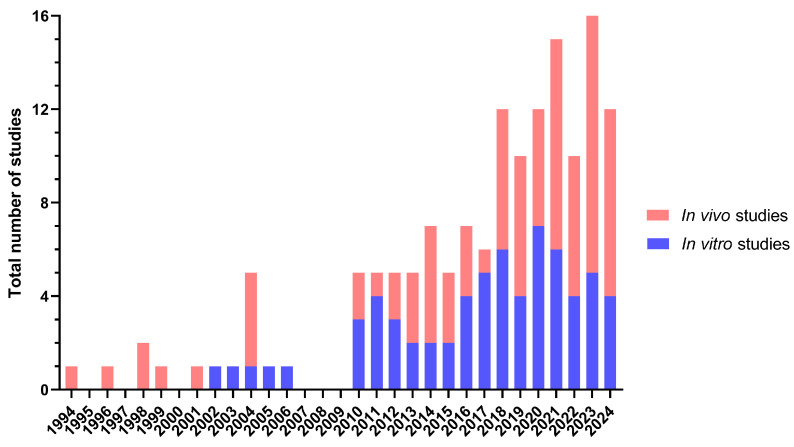
Number and type of preclinical studies reviewed and year of publication (latest search 4 August 2024).

In this review, 147 studies were classified as preclinical, where 66 were exclusively in vitro studies and 81 included an in vivo (or ex vivo) analysis (Figure 4).

#### 3.1.1. Animal Biomaterial Source

The use of blood plasma or its derivatives for TESS manufacture and other dermatological treatment strategies has been widely studied for more than 20 years at the preclinical level (Figure 4). Regarding the biomaterial source, most of the studies reported its use from a human origin [16,29,32,33,34,35,36,37,38,39,40,41,42,43,44,45,46,47,48,49,50,51,52,53,54,55,56,57,58,59,60,61,62,63,64,65,66,67,68,69,70,71,72,73,74,75,76,77,78,79,80,81,82,83,84,85,86,87,88,89,90,91,92,93,94,95,96,97,98,99,100,101,102,103,104,105,106,107,108,109,110,111,112,113,114,115,116,117,118,119,120,121,122,123,124,125,126,127,128,129,130,131,132,133,134,135,136,137], although bovine [34,37,48,138,139,140,141,142,143,144,145,146,147,148,149,150,151,152,153,154,155,156,157,158], rat [124,159,160,161,162,163,164,165,166], canine [57,167,168], porcine [86,169], ovine [170,171], goat [172] and rabbit [173] sources have also been evaluated (Table 1). Three of the in vivo studies reviewed did not indicated the source [174,175,176] but in all of them, the biomaterial was applied as a wound dressing, alone [175] or combined with others [174,175,176]. The target was the treatment of excisional wounds on rats [174] or mice [175], but also acute infected wounds [176], demonstrating a decrease in the wound inflammation levels and improving the healing process.

Interestingly, the number of studies comparing two or more sources was lower [34,37,48,57,86,124], which could be explained by the preference to use human source for clinical purposes. Among them, one in vitro study compared commercial human fibrinogen/fibrin and bovine fibrinogen for the manufacture of monolayer TESSs composed of human fibroblasts [37]. Results demonstrated similar abilities in inhibiting lattice contraction regardless of the source [37]. The other comparative investigations were in vivo research, where the human origin was compared with bovine [34,48], canine [57] or porcine [86] sources. In these, the biomaterial was used as a sealant [34,48], wound dressing [86] or as a pre-treatment before mesh skin grafting [57]. Results revealed that better scores in all clinical variables [57] and less inflammation [34] were achieved when human source was used, which supports that most of the preclinical studies have been focused on this origin.

#### 3.1.2. Type of Dermatological Treatment Approaches Manufactured and Wound Healing Purposes

**Figure 5:**
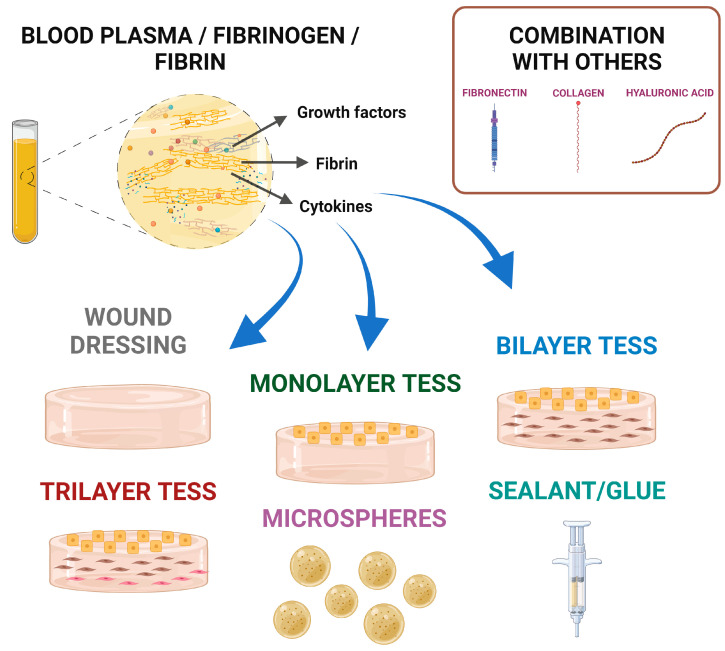
Dermatological treatment approaches developed using blood plasma, fibrinogen or fibrin as a biomaterial. *Created in BioRender. Sierra-Sánchez, Á. (2025) https://BioRender.com/h40k964*.

Regarding the role of blood plasma/fibrinogen/fibrin for the development of dermatological treatment approaches, it has been demonstrated to be versatile as a scaffold with or without cells (Figure 5).

Considering the number of studies (Supplementary Table S1), the biomaterial has been mainly used for the manufacture of wound dressings, especially in vivo [29,57,59,65,71,80,83,86,89,90,91,92,97,104,108,109,115,117,118,121,122,126,128,129,138,139,143,149,153,156,158,159,160,163,166,167,168,174,175,176] than in vitro [36,43,98,99,116,120,130,134], together with the development of monolayer TESSs (one cell type) comprised of dermal [16,37,64,72,74,76,82,84,85,87,90,91,100,101,105,120,140,144,145,146,148,152,154,156,169,170], epithelial [35,38,44,45,64,87,90,105,123,161,170,173] or mesenchymal stem cells (MSCs) [54,60,66,70,81,87,94,97,127,128,132,146,147,150] from human or animal origin. This could be explained because of the difficulties associated with the development of more complex strategies where more aspects such as viability, hydrogel degradation rate or bio-integration could be challenging. Despite of this, the fabrication of bilayer TESSs (two cell types) has been widely studied, combining mainly epithelial and dermal cells, [29,33,39,40,41,42,45,47,52,55,56,63,69,72,77,88,93,101,102,105,106,110,111,112,119,121,124,125,131,136,137,164,170,171]. However, fibroblasts have also been evaluated together with endothelial cells [75,113,155], MSCs [58] or MSCs differentiated into endothelial cells and pericytes [49], demonstrating the importance of achieving a well-formed dermal layer for dermatological purposes.

Moreover, trilayer TESSs (three cell types) have also been analyzed. In most of these studies, epidermal and dermal cells were combined with MSCs [61,67,96,142] or endothelial cells [95,103,141] to resemble a hypodermal layer. Interestingly, one study evaluated the use of MSCs differentiated into the three layers of the skin to develop TESSs that could be utilized for immediate wound coverage without the need for cell expansion [53]. In addition, other research compared three different stromal layers; (i) endothelial cells and fibroblasts, (ii) MSCs and fibroblasts and (iii) endothelial cells, MSCs and fibroblasts which were cultured with keratinocytes on top [68]. After in vitro analysis, the formation of a skin substitute with similar structure to native skin was demonstrated in all cases, but the release of angiogenic factors was increased when endothelial and MSCs were included [68].

Finally, other wound healing strategies or applications such as a carrier for microspheres or spheroids of MSCs [79] or MSCs and fibroblasts [114] have been developed. In these, the use of fibrin hydrogels increased the growth factor secretion [79] and triggered cell migration [114]. Moreover, the development of sealants or glues for in vivo purposes have been explored [32,34,48,62,133,162], demonstrating better results than the use of standard/control treatments with sutures or autoclips in terms of blood vessel formation [32], inflammatory response (less) [34], tissue integrity [48] and epithelialization [162]. Interestingly, one of these studies analyzed the concentration of thrombin into two commercially fibrin biomatrices demonstrating that the lower concentration (4 IU/mL) generated more functional vessels in an excisional wound model [62]. To conclude, other applications of this biomaterial have been developed such as bioinks for bioprinting [73], sprays for topical application [157] and for subcutaneous injection in different skin wound models, alone [78,163,165] or with cells [113,151], demonstrating its versality.

#### 3.1.3. Skin Wound Models Studied In Vivo

In addition to the analysis of the biomaterial source and the type of TESSs and strategies developed, in the specific case of the in vivo studies (81), the type of skin wound treated and the animal models used were analyzed (Table 2).

Excisional or surgical wounds were the main type of injuries analyzed [29,32,33,34,35,38,39,41,46,48,51,56,57,58,59,60,62,65,69,71,80,89,91,92,94,97,104,108,109,121,124,126,128,129,133,137,138,139,141,142,150,151,153,155,156,158,159,160,162,164,166,167,168,170,171,173,174,175,176] using several animal models such as pigs [109], mice [29,32,33,35,38,39,41,46,51,56,58,59,65,69,80,92,104,108,121,129,137,138,139,151,153,155,156,175], rats [48,60,62,71,91,94,97,124,126,128,141,142,150,158,159,160,162,164,166,173,174,176], dogs [34,57,168], cats [167], rabbits [133], sheep [170,171] or even, human ex vivo skin wound models [89]. Regarding the use of the blood plasma and its derivatives, the wound dressing strategy was preferred [29,57,59,65,71,80,89,91,92,97,104,108,109,121,126,128,129,138,139,153,156,158,159,160,166,167,168,174,175,176], followed by bilayer [29,33,39,41,56,58,69,121,124,137,155,164,170,171] and monolayer [35,38,46,51,60,91,94,97,128,150,156,170,173] TESSs. Moreover, its use as a sealant/glue [32,34,48,62,133,162], for trilayer TESSs manufacture [141,142] and as a carrier for direct injection of mice skin-derived precursors and epidermal stem cells into the wound [151] have also been reported. Interestingly, in seven of these studies, two treatment strategies were compared: wound dressing vs. monolayer TESSs on rats [91,97,128] and mice [156], wound dressing vs. bilayer TESSs on mice [29,121] and monolayer vs. bilayer TESSs on sheep [170]. These studies revealed that the combination of human MSCs (hMSCs) with commercial fibrin [128] or pegylated commercial human platelet-free plasma (PFP) [97] increased wound closure, re-epithelialization (confirmed by Keratin 10 and 14 immunofluorescence staining), neovascularization [128] and blood vessel density [97] compared to wound dressing strategy, so the addition of human cells improved the wound healing process. Moreover, the combination of human plasma with human fibroblasts and keratinocytes (bilayer TESSs) also demonstrated a proper clinical integration and epithelization [29,121], better than wound dressings. Comparison of monolayer and bilayer TESSs manufactured with ovine plasma and combined with ovine fibroblasts and keratinocytes, demonstrated that bilayer TESSs-treated wounds achieved complete epithelialization after 21 days, whereas this was incomplete when monolayer TESSs were applied [170]. Finally, another study compared the use of photo-crosslinked bovine fibrinogen tissue sealant, a commercial human-based fibrin wound glue and wound clip closures [48]. A proper rate and degree of wound healing and excellent tissue integrity was achieved using the bovine sealant and comparable to the human glue and standard wound clip closures [48].

A complete comparison and analysis of the effect of the biomaterial for the development of preclinical strategies for the treatment of excisional or surgical wounds is extremely complicated. As can be observed (Table 2), several non-cellular and cellular strategies have been analyzed, together with a wide range of animal models. However, the conclusion is that the main concern and application of this biomaterial at the preclinical level, seems to be focused on the treatment of excisional/surgical wounds due to their prevalence in a clinical environment.

Regarding the number of studies, the second model of injury analyzed have been the burns [70,81,86,117,118,147]. In these, only two treatment strategies have been evaluated: wound dressings [86,117,118] and monolayer TESSs [70,81,147]. The animal model preferred was the use of pigs, whose skin is more similar to human skin [177]. These studies revealed interesting results such as the application of pegylated autologous platelet-free plasma as a wound dressing for the treatment of thermal injuries, which promoted a slower deposition of the granulation tissue through the time compared to pegylated commercial fibrin hydrogels [86]. Moreover, this strategy did not reduce the number of immune cells infiltrated within the granulation tissue, due probably to the limited overall concentration of fibrinogen within the platelet-free plasma gel [86]. Furthermore, the combination of fibrin with other biomaterials such as silk fibroin and hyaluronic acid [117] or hyaluronic acid and synthetic polymers [118] for the development of wound dressings, demonstrated to promote burn healing through a mature epithelium regeneration in rabbits, and in a better level than the use of commercial matrix treatments [117,118]. In those studies where monolayer TESSs composed of MSCs were manufactured, the inclusion of the cells into the hydrogel improved wound vascularization and re-epithelialization in rats [70] or pigs [81,86].

Other skin wounds such as chronic [80,115,143,157] and several types of ulcers [83,107] have also been treated with wound dressings constituted of blood plasma, fibrinogen or fibrin biomaterial [80,83,115,143,157] or through intradermal injection [107]. In those cases where wound dressings were applied for chronic wounds, the biomaterial was combined or functionalized with other molecules such as laminin heparin-binding domains [80], bismuth oxychloride [157], nanoparticles loaded with epidermal growth factor (EGF) [143], or platelet exosomes [115], resulting in an increased wound healing rate in mice [80,157], porcine skin [143] or rabbits [115]. In the case of ulcers, a too high human platelet concentration (10^7^ platelets/µL) for the manufacture of wound dressings seemed to reduce the hydrogel resorption in mice due to the promotion of an increased granulation and adipose tissue formation and angiogenesis [83]. However, both low (2 × 10^6^ platelets/µL) and high platelet concentration increased the thickness of the regenerated epidermis compared to untreated wounds, indicating that the lower could be more useful for the treatment of superficial skin lesions [83]. Interestingly, the intradermal injection in mice of a commercial human plasma combined with growth factors promoted effective arteriogenesis in the dermal layer [107].

Moreover, the use of blood plasma as a wound dressing or injection has been applied over severe radiation-induced skin injuries, demonstrating that both strategies could reduce inflammation and promoting angiogenesis and vascular regeneration [163]. However, injected plasma had a higher concentration of platelets and platelet-derived growth factors due to the preparation method and, therefore, better repair effect was achieved [163].

Apart from these skin wound models, blood plasma and its derivatives have been evaluated in vivo to determine their safety, integration and angiogenic properties by subcutaneous implantation [90,127,149] or injection in mice [78,113] or rats [165]. In these, the biomaterial was evaluated alone [127,165] and combined with others [78,90,113,149] and with cells [113,127], promoting the microvascular network formation in the subcutaneous tissue [78], useful for improving wound healing and regeneration rates.

Finally, four studies evaluated the use of commercial fibrin [66,114] or platelet-rich human plasma [93,122] applying the chicken embryo chorioallantoic membrane (CAM) assay. This is an intermediate step in between the in vitro and the in vivo models that enables determining the angiogenesis properties and the material-tissue biocompatibility of the different conditions analyzed [178]. Applying this methodology, wound dressings [93,114,122], monolayer TESSs with human adipose tissue-derived MSCs (hAT-MSCs) [66] and bilayer TESSs composed of human umbilical cord blood MSCs (hUCB-MSCs) and fibroblasts [114] were analyzed. The results revealed that the combination of this biomaterial with others such as porous collagen-glycosaminoglycan [93] or gelatin methacrylate [122] increased angiogenic and vascularization potential [93,122] and, moreover, the addition of cells also stimulated the production of endogenous ECM [114] and the release of proangiogenic and cytokine factors [66].

#### 3.1.4. Biomaterials Combined with Blood Plasma, Fibrinogen or Fibrin for the Development of Dermatological Treatment Approaches

The blood plasma, fibrinogen or fibrin biomaterial has been mainly used alone for the development of the different dermatological treatment approaches reviewed, in vitro [16,36,40,42,43,44,45,50,55,61,64,67,68,72,75,76,79,95,96,98,99,102,110,111,112,116,120,123,130,131,135,140,144,146,169] or in vivo [32,33,34,35,38,39,41,46,48,51,57,62,65,66,69,78,80,83,90,92,104,107,108,114,115,121,127,128,129,133,137,139,141,142,143,150,151,153,157,159,160,163,164,165,167,168,170,171,175] (Supplementary Table S2). However, due to the nature of this biomaterial, the manufactured hydrogel could have a lack of mechanical resilience and its manipulation be complicated. Therefore, other biomaterials have also been combined with it to achieve a synergistic effect and improve its rheological aspects [106,116,120] but also biological aspects such as cell proliferation [146], cell survival rate [52] or wound healing properties [29,93,118,124].

A total of forty-four biomaterial combinations have been reported (Supplementary Table S2), with agarose [29,56,58,75,77,88,100,101,114,119,120,132], polyethylene glycol (PEG) [70,81,82,86,89,94,97,113,140,149], collagen [37,43,52,71,125,131,136,138,146], polylactic and polyglycolic acids [65,74,85,161,162,164] and hyaluronic acid [29,63,126,131,139,147] the most studied. For in vitro purposes, agarose [75,77,88,100,101,119,120,132] and collagen [37,43,52,125,131,136,146] have been the most investigated; however, for in vivo purposes, PEG [70,81,86,89,94,97,113,149], agarose [29,56,58,114] and hyaluronic acid [29,126,139,147] were the preferred biomaterials combined.

Among them, several studies directly compared the use of blood-plasma-derived biomaterial alone and combined with others. These biomaterials were agarose [75,120], collagen [43,131,146], elastin [112], PEG [140], graphene oxide [130] and silica or silica/chitosan [116]. In them, the results revealed that the combination with agarose is a useful strategy for the development of 3D bioprinting technologies [75] and, moreover, that an appropriate concentration of this secondary biomaterial is essential to increase the stiffness of the human plasma but maintained a good cell proliferation rate [120]. This was also observed when elastin was combined with plasma-based hydrogels, being more effective for improving mechanical properties and maintaining cell proliferation when the concentration was between 1–3 wt.% [112]. The rest of the comparative in vitro studies corroborated that the addition of biomaterials increased mechanical properties of fibrin-based hydrogels [43,116,140,146] or, even, improved the release of antibiotics through the time by allowing a sustained delivery over a prolonged period [130], which is relevant for wound healing purposes. Interestingly, another study compared two modifications of a fibrin-based scaffold with alginate or alginate/polymerized polydimethylsiloxane, demonstrating that the second conformation provided a significantly higher shear or storage modulus making it stronger but remaining fibroblast viability, proliferation and infiltration [145].

In the case of the comparative in vivo studies, most of the investigations evaluated the use of this blood plasma-derived biomaterial as a wound dressing [65,80,90,92,139,153,157,160,175]. Only one study compared the application of human platelet-rich plasma (hPRP) alone and combined with gelatin for subcutaneous injection, demonstrating an increased formation of capillaries and microvascular network in mice when gelatin was added [78].

Regarding the wound dressing strategy, the excisional/surgical wounds on mice was the preferred model [65,80,92,139,153,160,175]. In them, the acellular dermal matrix (ADM) [92], the polylactic acid [65], the laminin heparin-binding domains [80], hyaluronic acid [139], the fibronectin [139], the sildenafil citrate [160], the polyvinyl alcohol [153] and rose-derived exosome-like nanoparticles (ELNs) [175] were tested. The results revealed that the combination of fibrin with polylactic acid provoked a faster wound healing process as well as dermal and epidermal regeneration [65] or that the addition of laminin heparin-binding domains improved the retention of growth factors such as VEGF or FGF and their efficiency in promoting wound healing [80]. Moreover, the addition of sildenafil citrate was more effective for wound healing, collagen remodeling and epithelialization than plasma alone [160] and the addition of polyvinyl alcohol also increased angiogenesis, demonstrated by a slightly higher expression of CD31 and α-SMA markers [153]. In addition, the combination with ELNs reduced inflammation at the wound sites, compared to the use of fibrin gels alone, demonstrated by a decrease in TNF-α, IL-1β, IL6 and CXCL1 mRNA levels [175].

To conclude, two studies compared several biomaterial combinations for the treatment of surgical/excisional wounds on rats [174] and mice [29]. In one case, the wound dressings manufactured with fibrin and gelatin or gelatin/2-hydroxyethyl methacrylate (HEMA) or gelatin/2-hydroxypropyl methacrylate (HPMA) revealed that faster wound healing was achieved with fibrin-HEMA combination, due probably to the presence hydrophilic groups which indirectly enhances the synthesis of substances that stimulate the proliferation of the fibroblasts [174]. In the other study, bilayer TESSs (human fibroblasts and keratinocytes) and wound dressings manufactured with human plasma and hyaluronic acid or agarose were evaluated, demonstrating that TESSs reported better results in terms of homeostasis restoration and re-epithelialization [29]. Interestingly, in the case of the use of hyaluronic acid as secondary biomaterial, angiogenesis was increased and, therefore, better wound healing was achieved at a level comparable to the gold-standard treatment with autografts [29].

#### 3.1.5. A Specific Analysis of the Human Blood Plasma, Fibrinogen or Fibrin as a Biomaterial and Its Combination or Not with Human Cells for Preclinical Dermatological Treatment Approaches

Several animal (non-human) sources have been reported and investigated for the development of preclinical strategies based on the use of blood plasma, fibrinogen or fibrin (Table 3). However, it is well known that the application of animal origin products presents immunogenic potential, biological variability, and raises ethical concerns about animal welfare [179].

Therefore, the use of this biomaterial from human origin is the preferred strategy (Table 4) mainly when the final purpose is its translation into a clinical environment. Also, for this reason, when cells are combined with this biomaterial for the development of tissue-engineered strategies, human cells are the most investigated.

Regarding the role of human blood plasma, fibrinogen or fibrin for the development of dermatological treatment approaches, its versatility as a scaffold with or without cells has been demonstrated (Table 4). Among them, the wound dressing approach as a temporary or permanent treatment for wounds has reported positive results in terms of dermal repair and vascularization in vivo [108]. In addition, the combination of these wound dressings with other components such as laminin heparin-binding domains [80] or ADM [92] increased its regenerative potential and promoted wound healing in vivo.

However, the combination with human cells reported better results of wound closure, re-epithelialization and neovascularization compared with the wound dressing strategy [128]. For this reason, totally human monolayer, bilayer and trilayer TESSs have been widely studied.

As previously indicated (Supplementary Table S1), monolayer TESSs have been extensively studied at the preclinical level and several wound in vivo models have been evaluated. Regarding the human cell type used and how biomaterial and cells were combined, different strategies have been reported; for example, human keratinocytes have been embedded into human plasma hydrogels [64,123], although the most usual is to culture them on top [35,38,44]. On balance, the use of human fibrin as a scaffold for keratinocytes growth has shown proper adhesiveness and survival rates especially when the content of fibrinogen is lower (15%) [87]. Regarding their wound healing potential, the engraftment of these monolayer TESSs on mice with full-thickness skin defects reported good take percentage after 2–3 weeks and expression of type IV collagen, a key protein of the basement membrane was confirmed [35]. On the other hand, when human fibroblasts were used for monolayer TESS manufacture, they have usually been embedded into the hydrogel, although some mechanical studies explored culturing them on top [16]. However, the encapsulated fibroblasts have shown good proliferation rates, either clinical fibrin or plasminogen-depleted fibrin were used [64] and provided good elastic properties [105]. Moreover, the use of these monolayer substitutes for regenerative purposes has been proved over surgical skin defects on diabetic rats, reporting higher re-epithelialization and collagenization rates compared to controls without cells [91].

Apart from human keratinocytes and fibroblasts, other cell types such as hMSCs [54,60,66,94,97,127,128,132] and perivascular [46] or endothelial cells [50] have been studied embedded into [46,60,94,97,128,132] or cultured over [50,66,127] human fibrin hydrogels. Interestingly, most of the studies reporting the use of hMSCs have applied hAT-MSCs [60,66,94,97,127], but cells from bone marrow (hBM-MSCs) [54], hUCB-MSCs [128] and Wharton’s jelly-derived MSCs [132] have also been evaluated, although there is a lack of studies that compare them directly. Regarding the application of these cellular TESSs for wound healing purposes, most of the in vivo studies have been focused on the angiogenic properties, demonstrating that neovascularization was achieved in full-thickness wounds on mice or rats [128]. This was accomplished by promoting faster angiogenesis and vascular regeneration [127] than control treatments without cells [94], which also improved overall re-epithelialization [46].

Despite the successful results observed with human monolayer TESSs, the incorporation and combination of different cell types to develop more complex skin substitutes have provoked the study of the human bilayer TESSs. Most of these substitutes are constituted of human fibroblasts embedded into a hydrogel as a dermal component and then human keratinocytes are seeded and cultured on top. However, other cell combinations have also been reported, such as human endothelial cells and fibroblasts embedded into a pegylated fibrin matrix and injected subcutaneously into mice [113]. In addition, the culture of human Wharton’s jelly-derived MSCs as epidermal layer over a dermal layer of plasma/agarose and human fibroblasts, demonstrated their capacity to in vivo differentiate into epithelial cells, developing another promising type of substitutes [58].

However, the combination of human fibroblasts and keratinocytes is preferred for bilayer TESS development, in vitro [40,42,45,55,77,88,93,101,105,106,110,111,112,125,131,136] or in vivo [29,33,39,41,56,69,121,137]. Regarding the in vitro studies, these demonstrated the feasibility of developing and culturing a well differentiated epidermis [40,42,131,136]. To achieve a proper characterization, their optical properties have also been studied with similar results in terms of absorption and scattering to the native human skin [101]. Moreover, the concentration of fibrin and the contraction of hydrogels were also studied, concluding that a higher concentration of fibrin is able to reduce contraction [110], although the hydrogel showed a height reduction of 30% that is unavoidable during the first 24 h likely due to the intrinsic fibrin matrix properties [111]. In addition, the possibility of storing these bilayer TESSs at 4 °C in basal medium for 3 days has been corroborated [55], which could be useful when several subsequent surgeries are required. Finally, the development of innervated skin has been reported, exhibiting nociceptor markers that resemble their native counterpart [125].

In the case of in vivo studies, most of the bilayer TESSs were studied in full-thickness or surgical skin wounds generated on mice. Applying this strategy, a stratified and keratinized epithelium was observed [33], basement membrane formation was corroborated by a correct expression of laminin at the dermal-epidermal junction [41] and a proper development of the dermis was also reported [56], resembling native human skin structure. Interestingly, this model has also been used for the development of skin substitutes constituted of transduced keratinocytes with type VII collagen for the treatment of recessive dystrophic epidermolysis bullosa [39]. Results demonstrated the generation of a cohesive and orderly stratified epithelium with all the characteristics of normal human epidermis, including rapid formation of anchoring fibrils [39].

In recent years, the development of human blood plasma-based trilayer TESSs has increased, mainly for in vitro studies. As in the case of the bilayer substitutes, human fibroblasts and keratinocytes are preferred for dermis and epidermis formation. However, for the hypodermis-like layer, hMSCs from different sources [61,67,68], human adipocytes [67] or human endothelial cells [68,95] have been studied.

Among hMSCs, hAT-MSCs were the most studied, demonstrating their capacity to promote the production of ECM that stimulates cellular growth, proliferation and keratinocyte differentiation [61,67,68]. Moreover, these cells were able to differentiate into adipocytes, when cultured together with mature adipocytes [67], or endothelial cells, promoting angiogenesis and skin repair and regeneration by releasing VEGF or hepatocyte growth factor (HGF) [68]. Interestingly, one study used hAT-MSCs to be differentiated into epithelial cells, fibroblasts and adipocytes when mixed with the appropriate scaffolds (pegylated fibrin and collagen) and inducers, demonstrating their suitability for the development of trilayer TESSs utilized for immediate wound coverage [53].

On the other hand, few in vivo studies on rats have reported the use of trilayer TESSs but using bovine fibrin to develop the hydrogel [141,142]. Results demonstrated that the addition of hAT-MSCs as hypodermal layer anastomosed to the recipient’s vasculature within only four days and the neo-epidermis efficiently established tissue homeostasis, without contraction of the dermis [142]. Despite the positive outcomes observed using trilayer TESSs, the lack of in vivo studies demonstrates the necessity of further research.

On balance, the number of preclinical studies evaluating the use of human origin of the blood plasma, fibrinogen or fibrin and cells is high (Table 4), which has led to their translation for the development of clinical treatments.

### 3.2. Clinical Use of Human Blood Plasma, Fibrinogen or Fibrin for Dermatological Treatment Approaches

**Figure 6:**
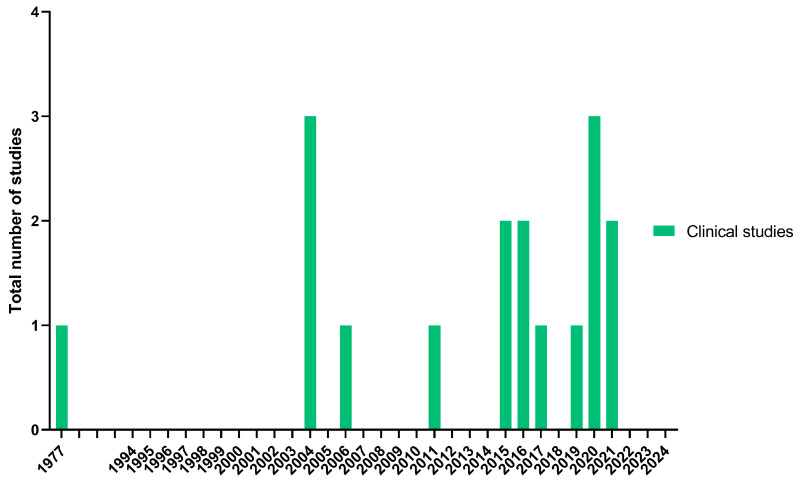
Number of clinical studies reviewed and year of publication (latest search 4 August 2024).

The translation of any treatment from preclinical to clinical studies is a difficult step due to the safety, effectiveness, quality and economical necessities required, and even more, when advanced therapies are developed. In the case of the human plasma, fibrinogen or fibrin, despite its skin wound healing properties, the molecular mechanisms of lateral aggregation and branching in its polymerization have not been completely and mechanistically identified. Moreover, most of the preclinical research has been on mice models; thus, much remains unknown about their roles in other mammals in the short and long term [180]. For these reasons, the number of clinical studies exploring the use of this biomaterial for dermatological treatments is limited (Figure 6): 12 case series or reports [181,182,183,184,185,186,187,188,189,190,191,192], 3 clinical trials [193,194,195], 1 observational study [196] and 1 pre–post pilot study [197]. Among them, one study is a protocol description [193] and the other two have already recruited patients, but no results have been posted yet [189,194] (Table 5).

Regarding tissue source, autologous [184,187,190,191,192,193,197] or allogeneic [181,182,183,185,186,188,189,195,196] origin has been evaluated indistinctly for clinical purposes. Wound dressings without cells [181,182,187,190,191,192,193] and bilayer TESSs [183,185,186,188,189,195,196] have been the preferred strategies to study, followed by monolayer [184,194,197] and trilayer TESSs [195]. Interestingly, hMSCs from allogeneic [184,194] or autologous [197] sources were the only type of cells used for monolayer TESSs manufacture, while for bilayer and trilayer TESSs, autologous cells such as keratinocytes, fibroblasts [183,185,186,188,189,195,196] and hAT-MSCs [195] for epidermal, dermal and hypodermal layers respectively, were preferred.

Moreover, three studies used this biomaterial as a temporary cover before skin grafts were applied [182,187,194]. In addition, several types of wounds have been treated such as periorbital skin defects [181], chronic wounds [182,191], ulcers [187,193,195,197] or traumas [184,192]. However, burns have been the most studied in terms of number of studies and patients [183,185,188,189,194,196].

To conclude, the use of human blood plasma, fibrinogen or fibrin alone has been the preferred strategy [181,183,184,185,186,187,188,191,192,194,196,197], but other biomaterials or scaffolds such as Integra^®^ [182], gelatin or hydrocolloids dressings [193], agarose [189], human collagen [195] or human amnion [190] have been combined to improve its mechanical and regenerative properties.

## 4. Discussion

Skin wounds or injuries can be minor, and therefore not serious, so if we care for them properly, the physiological wound healing process is able to resolve them. However, when this process is deregulated for several factors or when large size wounds are generated, this poses a big problem for the patient and for the global public health system. For example, burns provoke an estimated 180,000 deaths annually and the mean total healthcare cost per burn patient is USD 88,218 (2014) [198].

For these reasons, the development of safety and efficient treatments is essential to solve or reduce these possibilities. In this context, the research of new therapies based on the use of biomaterials (alone or combined with cells) is a promising approach. In this systematic review, it has been demonstrated that the research of the blood plasma, fibrinogen or fibrin is an interesting strategy due to the intrinsic biological properties of the biomaterial. At the preclinical level, a high number of studies have reported its use, especially from human origin and without being combined with secondary biomaterials. Moreover, most of the in vivo studies have evaluated their use as a wound dressing due to the content of growth factors and molecules that improve wound healing. However, a relevant number of studies have also evaluated its combination with one or two cell types (monolayer and bilayer TESSs respectively) to increase their regenerative potential for excisional/surgical wounds and burns, mainly.

The revision of the preclinical studies demonstrated that there is a lack of standardization in terms of manufacture or preferred strategies. Firstly, even in the case of the less studied sources, several variations in the biomaterial have been explored, such as blood plasma from different donors, and their associated variability, commercial fibrinogen or fibrin, platelet-rich plasma or plasminogen-depleted fibrin. This means that there is not still a perfect or promising strategy that takes advantage above the others. Moreover, the application of many other biomaterials combined with the blood plasma or its derivatives to improve its mechanical properties demonstrates that this aspect should be addressed to find a promising approach to focus. However, this determines the versatility of the biomaterial to be used under different conditions and circumstances, which is also interesting. Finally, the release of the growth factors associated with blood plasma should be also investigated and evaluated, but due to the variability in the sources and donors, it is difficult to determine a standard composition. In addition, the application of derivatives such as fibrinogen or fibrin entails the lack of these growth factors or molecules which makes it difficult to directly compare the studies.

Regarding clinical research, despite the large number of preclinical in vivo studies evaluating the use of this biomaterial for skin wound healing, its translation into a clinical environment is still limited. This could be explained by the fact that most of these studies are carried out in rodents, so more accurate animal skin models should be analyzed (pigs). In addition, the lack of standardization of the mechanical and biological properties due to the inter-variability between donors, could also play an important role that leads to this fact. However, among the clinical studies that exist, blood plasma, fibrinogen or fibrin from human origin, alone as a wound dressing or for the development of bilayer TESSs (human fibroblasts and keratinocytes) have been the preferred strategies, demonstrating their safety, and promoting epithelialization and epidermal regeneration, especially in severely burned patients.

In conclusion, the use of blood plasma/fibrinogen/fibrin as a biomaterial for the development of dermatological treatment approaches is a useful strategy as has been widely demonstrated in vitro and in vivo. However, although clinical studies have revealed its safety and wound healing potential, a higher number of them should be carried out in the coming years to increase the number of patients recruited and to evaluate a greater range of biomaterials and/or cells combinations, as well as more dermatological diseases.

## 5. Limitations and Future Directions

The limitation of this systematic review is the wide range of different uses and applications of blood plasma and its derivatives for the development of dermatological treatment purposes, which makes it difficult to compare the results. Moreover, this bibliographic search has been focused on those studies where this biomaterial has been used as a main component; however, its application could also be as a secondary component or to enhance the effect of other strategies or therapies. For these reasons, a future review should include the benefits, mainly for clinical purposes, as a complementary approach. Moreover, apart from its use for skin wound healing purposes, the use of fibrin seems to be interesting in the field of cosmetics or aesthetic interventions, which could increase the number of clinical studies analyzed to reinforce its application in dermatology.

## Figures and Tables

**Table 1:** Sources of blood plasma, fibrinogen or fibrin biomaterial investigated for the development of dermatological treatment approaches: type and number of studies reviewed.

Blood Plasma/Fibrinogen/Fibrin Source	In Vitro Studies/References	In Vivo Studies */References	Total Number of Studies	** Studies Evaluating Two Biomaterial Sources
Human	56	52	108	6
	[16,36,37,40,42,43,44,45,47,49,50,52,53,54,55,61,63,64,67,68,72,73,74,75,76,77,79,82,84,85,87,88,95,96,98,99,100,101,102,103,105,106,110,111,112,116,119,120,123,125,130,131,132,134,135,136]	[29,32,33,34,35,38,39,41,46,48,51,56,57,58,59,60,62,65,66,69,70,71,78,80,81,83,86,89,90,91,92,93,94,97,104,107,108,109,113,114,115,117,118,121,122,124,126,127,128,129,133,137]		
Bovine	8	16	24	
	[37,140,144,145,146,148,152,154]	[34,48,138,139,141,142,143,147,149,150,151,153,155,156,157,158]		
Rat	1	8	9	
	[161]	[124,159,160,162,163,164,165,166]		
Canine	0	3	3	
		[57,167,168]		
Porcine	1	1	2	
	[169]	[86]		
Ovine	0	2	2	
		[170,171]		
Goat	1	0	0	
	[172]			
Rabbit	0	1	1	
		[173]		
Unknown	0	3	3	
		[174,175,176]		
Total	67	86	153	
Total number of individual studies reviewed			153 − 6 = 147	
* Ex vivo studies and chorioallantoic membrane (CAM) assays are considered as in vivo studies ** This number of studies must be subtracted to the final total number of studies to know the number of individual studies reviewed				

**Table 2:** Type of skin wounds where blood plasma, fibrinogen or fibrin was in vivo investigated as a biomaterial; type of TESS or strategy manufactured and animal models used.

Type of Skin Wound	TESS/Strategy	Animal Model	Number of Studies/References	Total Number of Studies	* Studies Evaluating Two or More Conditions
Excisional/Surgical	Injection into the wound - 2 cell types	Mouse	1[151]	66	9
	Sealant/glue	Mouse	1[32]		
		Rat	3[48,62,162]		
		Dog	1[34]		
		Rabbit	1[133]		
	Wound dressing	Ex vivo – Human skin	1[89]		
		Pig	1[109]		
		Mouse	14[29,59,65,80,92,104,108,121,129,138,139,153,156,175]		
		Rat	11[71,91,97,126,128,158,159,160,166,174,176]		
		Dog	2[57,168]		
		Cat	1[167]		
	Monolayer	Mouse	5[35,38,46,51,156]		
		Rat	7[60,91,94,97,128,150,173]		
		Sheep	1[170]		
	Bilayer	Mouse	10[29,33,39,41,56,58,69,121,137,155]		
		Rat	2[124,164]		
		Sheep	2[170,171]		
	Trilayer	Rat	2[141,142]		
Burn	Wound dressing	Pig	1[86]	6	
		Rabbit	2[117,118]		
	Monolayer	Pig	2[81,147]		
		Rat	1[70]		
Chronic	Wound dressing	Ex vivo – Porcine skin	1[143]	4	
		Mouse	2[80,157]		
		Rabbit	1[115]		
Ulcer	Wound dressing	Mouse	1[83]	2	
	Intradermal injection		1[107]		
Radiated injury	Wound dressing and Injection	Rat	1[163]	1	
No wound – Subcutaneous implantation	Wound dressing	Mouse	2[90,149]	3	
	Monolayer		1[127]		
No wound – Subcutaneous injection	Without cells	Mouse	1[78]	3	
		Rat	1[165]		
	Two cell types	Mouse	1[113]		
Chorioallantoic membrane (CAM) assay	Wound dressing	Chicken embryo	3[93,114,122]	5	
	Monolayer		1[66]		
	Bilayer		1[114]		
TOTAL				90	
Total number of individual studies reviewed			90 − 9 = 81		
* This number of studies must be subtracted to the final total number of studies to know the number of individual studies reviewed					

**Table 3:** Preclinical studies reviewed, evaluating the use of the blood plasma, fibrinogen or fibrin from non-human source.

Reference / Year	Biomaterial Source	Combination with Other Biomaterials	Dermatological Treatment Approach Developed	Type of Study	Animal Model and Type of Wound	Cells Used	Outcomes
[138] / 1994	Commercial Bovine Fibrin	1-Collagen	Wound Dressing	In vivo	Mice/Surgical Wounds	-	The resulting fibroblast-infiltrated tissue resembled a normal dense connective tissue
[139] / 1996	Commercial Bovine Fibrin	1-Alone 2-Fibronectin 3-Hyaluronic Acid	Wound Dressing	In vivo	Mice/Surgical Wounds	-	The number of microvessels formed within fibrin-impregnated samples was increased in the presence of hyaluronic acid
[34] / 1999	Human and Bovine Fibrin	1-Alone	Sealant/Glue	In vivo	Beagle Dogs/Excisional Wounds	-	After 10 and 30 days, scars were larger using bovine sealant
[37] / 2003	Commercial Human and Bovine Fibrin	1-Collagen	Monolayer TESS	In vitro	-	Human Fibroblasts	Both sources decreased the contraction of fibroblast populated collagen lattices in a dose-dependent manner
[174] / 2004	Fibrin from Animal Blood	1-Gelatin 2-Gelatin/Hydroxyethyl methacrylate (HEMA) 3-Gelatin/Hydroxypropyl methacrylate (HPMA)	Wound Dressing	In vivo	Rats/Excisional Wounds	-	Complete wound healing was achieved in all cases
[48] / 2010	Commercial Human Fibrin (Tisseel) and Commercial Bovine Fibrin	1-Alone	Sealant/Glue	In vivo	Rats/Surgical Wounds	-	Fibrin groups showed excellent tissue integrity with signs of thick collagen fiber bundles throughout the tissue
[169] / 2011	Blood Plasma from Pigs	1-Alone	Monolayer TESS	In vitro	-	Dermal Fibroblasts from Pigs	Fibroblasts showed good adhesion and normal distribution
[57] / 2012	Commercial Human Fibrin (Tissucol^®^) and Dog Platelet-Rich Plasma	1-Alone	Wound Dressing	In vivo	Dogs/Surgical Wounds	-	Clinical evaluations showed that the human fibrin group showed better scores for all variables compared to dog platelet-rich plasma group
[140] / 2013	Commercial Bovine Fibrin	1-Alone 2-Polyethylene glycol (PEG)	Monolayer TESS	In vitro	-	Human Fibroblasts	PEG hydrogels exhibited cell-mediated stiffening concurrent with their dynamic morphogenesis, as indicated by a four-fold increase in storage modulus after 1 week in culture
[170] / 2014	Ovine Bood Plasma	1-Alone	Bilayer TESS (Fibroblasts and Keratinocytes) Monolayer TESS (Fibroblasts) Monolayer TESS (Keratinocytes)	In vivo	Sheep/Full-Thickness Wounds	Ovine Fibroblasts and Keratinocytes	Bilayer TESSs demonstrated the best healing potential
[171] / 2014	Ovine Blood Plasma	1-Alone	Bilayer TESS	In vivo	Sheep/Full Thickness Skin Lesions	Ovine Fibroblasts and Keratinocytes	Wounds treated with bilayer TESSs healed faster than control group
[141] / 2014	Commercial Bovine Fibrin	1-Alone	Trilayer TESS	In vivo	Rats/Surgical Full-Thickness Skin Defects	Human endothelial cells, Fibroblasts and Keratinocytes	The bioengineered human lymphatic capillaries were found to anastomose to the recipient rat’s lymphatic plexus as early as 14 days after in vivo grafting
[142] / 2014	Commercial Bovine Blood Plasma	1-Alone	Trilayer TESSs	In vivo	Rats/Surgical Wounds	Human AT-MSCs, Fibroblasts and Keratinocytes	Pre-formed vascular networks anastomosed to the recipient’s vasculature within only 4 days The neo-epidermis efficiently established tissue homeostasis
[143] / 2015	Commercial Bovine Fibrin	1-Alone	Wound Dressing	Ex vivo over porcine skin	Chronic Wounds	-	Fibrin-based scaffolds may be the most suitable approach to formulate growth factor-loaded lipid nanoparticles
[144] / 2016	Commercial Bovine Fibrin (Smart Matrix^®^)	1-Alone	Monolayer TESS	In vitro	-	Human Fibroblasts	The viscoelastic properties of Smart Matrix^®^ may be closer to those of human skin and dermis A higher cell proliferation was seen in the fibrin-based Smart Matrix^®^ compared to other scaffolds
[145] / 2017	Commercial Bovine Fibrin	1-Alginate 2-Alginate/Polymerized polydimethylsiloxane (Sil)	Monolayer TESS	In vitro	-	Human Fibroblasts	By adding the polymer, the resulting two-component scaffolds have a significantly higher shear or storage modulus G′
[86] / 2018	Commercial Human Fibrin and Blood Plasma from Pigs	1-Polyethylene glycol (PEG)	Wound Dressing	In vivo	Pigs/Burns	-	The treatment with the PEG-fibrin group displayed less contraction on day 28
[146] / 2018	Commercial Bovine Fibrin	1-Alone 2-Collagen	Monolayer TESS	In vitro	-	Human hBM-MSCs, Fibroblasts or Microvascular Endothelial Cells	The scaffolds supported excellent cell ingress and proliferation
[159] / 2019	Blood Plasma from Rats	1-Alone	Wound Dressing	In vivo	Rats/Surgical Wound	-	The blood plasma improved graft take and regulated the proliferation of a thicker and more uniform epidermis, while decreased healing time
[147] / 2020	Commercial Bovine Fibrin	1-Hyaluronic Acid	Monolayer TESS	In vivo	Pigs/Full-Thickness Burns	Human Wharton’s jelly-derived MSCs	Fibrin and mesenchymal stem cell sheets applied to the wound bed improved re-epithelialization, dermal cell repopulation, and neovascularization
[148] / 2020	Bovine Blood Plasma	1-Decellularized human skin-derived extracellular matrix (dsECM)	Monolayer TESS	In vitro	-	Human Fibroblasts	The hybrid hydrogel presented good rheological properties and shear thinning properties
[160] / 2020	Blood Plasma from Rats	1-Alone 2-Sildenafil citrate hydrogel (SCH)	Wound Dressing	In vivo	Rats/Excisional Skin Wounds	-	Combination of blood plasma and SCH treatment was more efficient in wound healing scoring, with less inflammation, more collagen remodeling, and more epithelization
[161] / 2020	Blood Plasma from Rats	1-Polylactic-co-glycolic acid (PLGA)	Monolayer TESS	In vitro	-	Human Keratinocytes	The scaffold demonstrated blood compatibility and improved cell adhesion and viability
[149] / 2020	Commercial Bovine Fibrin	1-Polyethylene glycol (PEG)	Wound Dressing	In vivo	Mice/Subcutaneous Implantation	-	Wound dressings were stable over 1-month following subcutaneous implantation, induced a minimal host inflammatory response, and displayed a substantial cellular infiltration and tissue remodeling
[162] / 2021	Commercial or Autologous Fibrin from Rats	1-Polyglycolic acid (PGA)	Sealant/Glue	In vivo	Rats/Surgical Epithelial Defects	-	Covering surgical wounds with autologous fibrin promoted better wound healing and epithelialization
[167] / 2021	Platelet-Rich Fibrin (PRF) from Dogs	1-Alone	Wound Dressing	In vivo	Cats/Surgical Wounds Or Abscess because of Dog Attacks	-	PRFs significantly induced healthy vascularized granulation tissue and also prompted the epithelization at the injured site
[150] / 2021	Commercial Bovine Fibrin	1-Alone	Monolayer TESS	In vivo	Rats/Surgical Wounds	Rat BM-MSCs	The fibrin gel containing BM-MSCs promoted wound healing and repair
[151] / 2022	Bovine Fibrin	1-Alone	Others: Injection	In vivo	Mice/Surgical Excision	Mice Skin-derived Precursors (SKPs) and Epidermal Stem Cells (Epi-SCs)	De novo hair genesis was observed in mice and the hairs persisted for a long time without teratoma formation. The blood vessels and sebaceous glands were also regenerated
[152] / 2022	Bovine Blood Plasma	1-Collagen/Alginate	Monolayer TESS	In vitro	-	Human Fibroblasts	The fibrous nature of the scaffold was characterized by high swelling properties and the quick release of calcium ions
[124] / 2022	Human Blood Plasma and Blood Plasma from Rats	1-Alginate/Gelatin	Bilayer TESS	In vivo	Rats/Full-Thickness Wounds	Rat Fibroblasts and Keratinocytes	Blood plasma facilitated vital physiological processes including ECM synthesis, macrophage polarization, and angiogenesis
[163] / 2023	Blood Plasma from Rats	1-Alone	Wound Dressing and Others: Injection	In vivo	Rats/Radiation-induced Skin Injuries	-	Blood plasma was capable of reducing inflammation and promoting angiogenesis and vascular regeneration
[164] / 2023	Blood Plasma from Rats	1-Alone 2-Polylactic-co-glycolic acid (PLGA)	Bilayer TESS	In vivo	Rats/Full-Thickness Excisional Wounds	Rat Fibroblasts and Keratinocytes	The bilayer TESSs induced granulation tissue growth, collagen deposition and epithelial tissue remodeling
[153] / 2023	Commercial Bovine Blood Plasma	1-Alone 2- Polyvinyl alcohol	Wound Dressing	In vivo	Mice/Full-Thickness Skin Wounds	-	The wound dressings were integrated and resorbed without inflammatory infiltration and promoted deeper neodermal formation, greater collagen fiber deposition and significantly accelerated wound healing and epithelial closure
[165] / 2023	Platelet-Rich Plasma (PRP) from Rats	1-Alone	Others: Injection	In vivo	Rats/Melasma	-	Four weeks after treatment with PRP, the skin of the rats was less pigmented and shiny than that of the control group
[154] / 2023	Bovine Fibrinogen	1-Collagen/Hyaluronic Acid/PEG succinimidyl glutarate (4S-StarPEG)	Monolayer TESS	In vitro	-	Human Fibroblasts	The resulting hydrogel exhibited promising properties as a scaffold, also facilitating the growth of and proliferation of the cells
[155] / 2023 (Bioprinting)	Commercial Bovine FIbrinogen	1-Gelatin methacrylate (GelMA)/Methacrylated Hyaluronic Acid (HAMA)	Bilayer TESS	In vivo	Mice/Full-Thickness Skin Wounds	Human Umbilical Vein Endothelial Cells (HUVECs) and Human Fibroblasts	The cell-seeded GelMA-HAMA-fibrin scaffold, under confined force loading, promoted neovascularization and wound restoration by enhancing blood vessel connections, creating a patterned surface, growth factors, and collagen deposition
[156] / 2024 (Bioprinting)	Commercial Bovine Fibrinogen	1-Gelatin methacrylate (GelMA)/Methacrylated Hyaluronic Acid (HAMA)	Wound Dressing and Monolayer TESS	In vivo	Mice/Full-Thickness Skin Wounds	Human Fibroblasts	3D bioprinted skin promoted epidermal regeneration, collagen maturation in the dermal tissue, and vascularization of the skin tissue to accelerate wound healing
[172] / 2024	Platelet-Rich Plasma (PRP) from Goats	1-Gelatin	Sealant/Glue	In vitro	-	-	Compared to commercially available fibrin glue, this showed comparable porcine skin adhesive strength at room temperature (∼40 kPa), with the added advantage of reduced swelling and slower degradation
[168] / 2024	Platelet-Rich Fibrin (PRF) from Dogs	1-Alone	Wound Dressing	In vivo	Dogs/Excisional, Surgical, Bites, Lacerations	-	PRF clots acted as a natural tissue filler, promoting epithelization and wound closure
[157] / 2024	Bovine Fibrinogen	1-Alone 2-Bismuth Oxychloride (BiOCl)	Others: Spray	In vivo	Mice/Chronic Diabetic Wounds	-	This approach improved the wounds resolution, the impaired angiogenesis, bacterial infection, and exacerbated inflammation of the chronic environment
[166] / 2024	Platelet-Rich Fibrin (PRF) from Blood Plasma from Rats	1-Pectin/Polyacrylic acid (Pec/PAA)	Wound Dressing	In vivo	Rats/Full-Thickness Skin Wounds	-	Wounds covered with tested hydrogel healed faster with more collagen deposition and re-epithelialization
[173] / 2024	Platelet-Rich Plasma (PRP) from Rabbits	1-Gelatin methacrylate (GelMA)/Methacrylated Hyaluronic Acid (HAMA)	Monolayer TESS	In vivo	Rats/Full-Thickness Skin Wounds	HaCaT	The biomimetic skin effectively facilitated early wound closure and rapid healing. PRP addition considerably reduced cell mortality following the peak of cell proliferation
[158] / 2024	Bovine Fibrin	1-Chitosan	Wound Dressing	In vivo	Rats/Full-Thickness Skin Wounds	-	Application of the wound dressing on open cutting out wounds increased cured rate with high biocompatibility than control treatments The experimental results showed virtually completed (97%) wound closure on the 12th day

**Table 4:** Preclinical studies reviewed, evaluating the use of the blood plasma, fibrinogen or fibrin from human source.

Reference / Year	Biomaterial Human Source	Combination with Other Biomaterials	Dermatological Treatment Approach Developed	Type of Study	Animal Model and Type of Wound	Cells Used	Outcomes
[32] / 1998	Commercial Human Fibrin	1-Alone	Sealant/Glue	In vivo	Mice/Excisional Wounds	-	No inflammatory reaction and an increased amount of blood vessels and capillaries was observed compared to a standard sealant
[33] / 1998	Human Blood Plasma	1-Alone	Bilayer TESS	In vivo	Mice/Surgical Wounds	Human Fibroblasts and Keratinocytes	A stratified, keratinized epithelium was reported, resembling the human epidermis
[34] / 1999	Human and Bovine Fibrin	1-Alone	Sealant/Glue	In vivo	Dogs/Excisional Wounds	-	After 10 and 30 days, scars were larger using bovine sealant
[35] / 2001	Commercial Human Fibrin	1-Alone	Monolayer TESS	In vivo	Mice/Excisional Wounds	Human Epithelial Cells	Application of human fibrin increased graft take percentage and the formation of a basement membrane was confirmed
[36] / 2002	Commercial Human Fibrin	1-Alone	Wound Dressing	In vitro	-	-	Fibrin decreased the length of the lag phase of keratinocyte activation and increased the consistency of the healing response
[37] / 2003	Commercial Human and Bovine Fibrin	1-Collagen	Monolayer TESS	In vitro	-	Human Fibroblasts	Both sources decreased the contraction of fibroblast populated collagen lattices in a dose-dependent manner
[38] / 2004	Commercial Fibrin from Human Blood Plasma	1-Alone 2-Integra^®^	Monolayer TESS	In vivo	Mice/Excisional Wounds	Human Keratinocytes	After 21 days, 83% of the grafted mice showed the presence of a differentiated human epidermis
[39] / 2004	Human Blood Plasma	1-Alone	Bilayer TESS	In vivo	Mice/Surgical Wounds	Human Fibroblasts and Keratinocytes	A cohesive and orderly stratified epithelia was observed after 2–6 weeks. A rapid formation of anchoring fibrils was reported
[40] / 2004	Commercial Human Fibrin	1-Alone	Bilayer TESS	In vitro	-	Human Fibroblasts and Keratinocytes	Fully differentiated stratified epidermis and basement membrane was formed after 15–17 days
[41] / 2004	Human Blood Plasma	1-Alone	Bilayer TESS	In vivo	Mice/Surgical Wounds	Human Fibroblasts and Keratinocytes	In the stable engrafted human skin, human laminin was localized at the dermo-epidermal junction
[42] / 2005	Commercial Human Fibrin	1-Alone	Bilayer TESS	In vitro	-	Human Fibroblasts and Keratinocytes	15 days of culture were optimal for the generation of keratinocyte layers with signs of differentiation
[43] / 2006	Commercial Human Fibrin	1-Alone 2-Collagen	Wound Dressing	In vitro	-	-	The elastic and viscoelastic characteristics of fibrin and collagen biomaterials can be determined reproducibly through compressive indentation experiments
[44] / 2010	Commercial Human Fibrin	1-Alone	Monolayer TESS	In vitro	-	Human Primary Keratinocytes or HaCaTs	Fibrin matrix promoted migration of keratinocytes to cover a larger area of culture
[45] / 2010	Commercial Human Fibrin	1-Alone	Bilayer TESS Monolayer TESS (Human keratinocytes)	In vitro	-	Human Fibroblasts and Keratinocytes	Cells maintained their proliferative potential and phenotype under the experimental conditions
[46] / 2010	Commercial Human Fibrin	1-Alone	Monolayer TESS	In vivo	Mice/Bilateral Full-Thickness Defects	Human Umbilical Cord Perivascular Cells (HUCPVC)	By 7 days, complete re-epithelialization of the wounds was observed
[47] / 2010	Commercial Human Fibrin	1-Alone 2-Collagen/Chitosan	Bilayer TESS	In vitro	-	Human Fibroblasts and Keratinocytes	The bilayer TESS had a histological structure like that of normal skin tissue
[48] / 2010	Commercial Human Fibrin (Tisseel) and Commercial Bovine Fibrin	1-Alone	Sealant/Glue	In vivo	Rats/Surgical Wounds	-	The use of fibrin as a glue showed excellent tissue integrity with signs of thick collagen fiber bundles throughout the tissue
[49] / 2011	Commercial Human Fibrin	1-Collagen/Polyethylene glycol (PEG)	Bilayer TESS	In vitro	-	Rat Adipose Tissue-derived MSCs	The fibrin characteristics can guide differentiation to endothelial and pericytes cells, confirmed by CD31 and von Willebrand factor expression
[50] / 2011	Commercial Human Fibrin	1-Alone	Monolayer TESS	In vitro	-	Human Endothelial Progenitor Cells (EPCs)	EPCs cultured on fibrin showed a significantly higher proliferation rate than over other scaffolds
[51] / 2011	Commercial Human Fibrin Fragment E	1-Alone	Monolayer TESS	In vivo	Mice/Excisional Wounds	Human Cord Blood-derived Endothelial Progenitor Cells (CB-EPCs)	Combination of Fibrin Fragment E and CB-EPCs accelerated wound closure and vascularization
[52] / 2011	Human Fibrin	1-Collagen	Bilayer TESS	In vitro	-	Porcine Fibroblasts and Keratinocytes	Use of human fibrin as a scaffold increased fibroblast survival
[53] / 2012	Commercial Human Fibrin	1-Collagen/Polyethylene glycol (PEG)	Trilayer TESS	In vitro	-	Human Adipose Tissue-derived MSCs	The use of this hydrogel induced an appropriate differentiation of the MSCs to immediate wound coverage
[54] / 2012	Platelet-Rich Plasma (PRP) from Human Blood Plasma	1-Integra^®^	Monolayer TESS	In vitro	-	Human Bone Marrow-derived MSCs	Seeded cells exhibited a greater aptitude to colonize the scaffold and showed an improved cell adhesion than compared to Integra^®^ alone
[55] / 2012	Human Blood Plasma	1-Alone	Bilayer TESS	In vitro	-	Human Fibroblasts and Keratinocytes	This bilayer TESSs can be stored in basal medium at 4 °C for at least 72 h before transplantation without compromising its functionality
[56] / 2012	Human Blood Plasma	1-Agarose	Bilayer TESS	In vivo	Mice/Surgical Wounds	Human Fibroblasts and Keratinocytes	A proper development of both dermis and epidermis was found after 30 days
[57] / 2012	Commercial Human Fibrin (Tissucol^®^) and Dog Platelet-Rich Plasma	1-Alone	Wound Dressing	In vivo	Dogs/Surgical Wounds	-	Clinical evaluations showed that the human fibrin group showed better scores for all variables compared to dog platelet-rich plasma group
[58] / 2013	Human Blood Plasma	1-Agarose	Bilayer TESS	In vivo	Mice/Excisional - Surgical Wounds	Human Wharton’s jelly-derived MSCs and Human Fibroblasts	In vivo grafting of the bioactive three-dimensional models demonstrated that hWJ-MSCs were able to stratify and to express typical markers of epithelial differentiation
[59] / 2013	Commercial Human Fibrin	1-Poly(ether)urethanepolydimethylsiloxane	Wound Dressing	In vivo	Mice/Full-Thickness Skin Wounds	-	Combination of the scaffolds with growth factors induced complete re-epithelialization, with enhanced granulation tissue formation/maturity and collagen deposition
[60] / 2013	Commercial Human Blood Plasma	1-Collagen/Polyethylene glycol (PEG)	Monolayer TESS	In vivo	Rats/Excisional Wounds model on rats	Human Adipose Tissue-derived MSCs	Combination of hAT-MSCs with the blood plasma scaffold induced differentiation to stromal and vascular phenotypes without the use of growth factors
[61] / 2013	Human Blood Plasma	1-Alone	Trilayer TESS	In vitro	-	Human Adipose Tissue-derived MSCs or Human Bone Marrow-derived MSCs, Human Fibroblasts and Keratinocytes	The inclusion of a subcutaneous layer contributed to an improved epidermal differentiation program
[62] / 2014	Commercial Human Fibrin	1-Alone	Sealant/Glue	In vivo	Rats/Excisional Wounds	-	Low concentration of thrombin generated more functional vessels and increased VEGF concentration after 7 days
[63] / 2014	Human Blood Plasma	1-Hyaluronic Acid	Bilayer TESS	In vitro	-	Human Fibroblasts and Keratinocytes	The human blood plasma provided the appropriate mechanical properties and cell adhesion sites to allow the tridimensional spreading of the human fibroblasts
[64] / 2014	Commercial Human Fibrin: Tisseel and Plasminogen-Depleted Fibrin	1-Alone	Monolayer TESS	In vitro	-	Human Fibroblasts or Keratinocytes	Human fibroblasts proliferated similarly in both types of fibrins, but keratinocytes proliferated less in plasminogen-depleted fibrin
[65] / 2015	Commercial Human Fibrin	1-Alone 2-Polylactic-co-glycolic acid (PLGA)	Wound Dressing	In vivo	Mice/Excisional Wounds	-	The combination of fibrin and PLGA resulted in much faster wound closure as well as dermal and epidermal regeneration
[66] / 2015	Commercial Human Fibrin	1-Alone	Monolayer TESS	In vivo	Chick Chorioallantoic Membrane (CAM) Assay	Human Adipose Tissue-derived MSCs	The combination of MSCs and fibrin resulted in an increased release of proangiogenic and cytokine factors
[67] / 2015	Commercial Human Fibrin	1-Alone	Trilayer TESS	In vitro	-	Human Adipose Tissue-derived MSCs or Human Adipocytes, Human Fibroblasts and Keratinocytes	The MSCs were able to differentiate into mature adipocytes during the course of four weeks and showed morphological resemblance to native adipose tissue. The keratinocytes formed and epithelial-like layer
[68] / 2015	Human Blood Plasma	1-Alone	Trilayer TESS	In vitro	-	Human Umbilical Vein Endothelial Cells (HUVECs), Human Adipose Tissue-derived MSCs, Human Fibroblasts and Keratinocytes	The MSCs secreted significant quantities of angiogenic and antiapoptotic factors that promotes angiogenesis and HUVECs proliferated and organized themselves into capillary-like structures
[69] / 2016	Human Blood Plasma	1-Alone	Bilayer TESS	In vivo	Mice/Full-Thickness Skin Defects	Human Fibroblasts Keratinocytes	The generated skin was very similar to human skin after 8 weeks
[70] / 2016	Commercial Human Fibrin	1-Polyethylene glycol (PEG)	Monolayer TESS	In vivo	Rats/Burns	Rat Adipose Tissue-derived MSCs	The application of PEG-fibrin biomaterial exhibited positive effects on granulation tissue formation. The use of MSCs improved vascularization of the injured area
[71] / 2016	Commercial Human Blood Plasma	1-Collagen	Wound Dressing	In vivo	Rats/Full-Thickness Skin Defects	-	The human collagen/blood plasma hydrogels accelerated the wound healing, angiogenesis and hair and sweat gland formation in vivo
[72] / 2016	Commercial Human Blood Plasma	1-Alone	Bilayer TESS Monolayer TESS (Rat Fibroblasts)	In vitro	-	Rat Fibroblasts and Keratinocytes	Co-culturing keratinocytes with fibroblasts in the human blood plasma constructs promoted fibroblast proliferation
[73] / 2016	Human Blood Plasma	1-Gelatin	Others: Bioink development	In vitro	-	3T3 Cell Line	The combination of human blood plasma and gelatin provided a natural scaffold for fibroblast-based bioink embedding and culture
[74] / 2016	Commercial Human Fibrin	1-Polylactic acid (PLA)	Monolayer TESS	In vitro	-	Human Fibroblasts	Fibrin stimulated the expression and synthesis of type I collagen in human dermal fibroblasts
[75] / 2017	Commercial Human Blood Plasma	1-Alone 2-Agarose	Bilayer TESS	In vitro	-	Human Umbilical Vein Endothelial Cells (HUVECs) and Human Fibroblasts	The hydrogel containing agarose and human blood plasma showed no capillary-like network formation after 14 days of culture
[76] / 2017	Human Blood Plasma	1-Alone	Monolayer TESS	In vitro	-	Human Fibroblasts	The structure and mechanical properties of the biomaterial allowed cell ingrowth and provided a sustained release of bioactive molecules
[77] / 2017	Human Blood Plasma from Diabetic and Healthy Patients	1-Agarose	Bilayer TESS	In vitro	-	Human Fibroblasts and Keratinocytes from Diabetic and Healthy Patients	Bilayer TESSs of diabetic patients showed reduced keratinocyte immunofluorescence intensity, but for fibroblasts and type IV collagen, greater intensity was reported
[78] / 2017	Platelet-Rich Plasma (PRP) from Human Blood Plasma	1-Alone 2-Gelatin	Others: Injection	In vivo	Mice/Subcutaneous Injection	-	PRP with gelatin hydrogels significantly promoted the formation of new capillaries and microvascular network in murine subcutaneous tissue
[79] / 2017	Commercial Human Fibrin	1-Alone	Microspheres	In vitro	-	Human Bone Marrow-derived MSC Spheroids	The microspheres stimulated endothelial cell proliferation, enhanced macrophage polarization and promoted angiogenesis
[80] / 2018	Commercial Human Fibrin	1-Alone 2-Laminin Heparin-binding domains	Wound Dressing	In vivo	Mice/Excisional Wounds Mice/Chronic Wounds	-	The incorporation of laminin heparin improved the retention of growth factors and enhanced he efficacy of them in promoting wound healing
[81] / 2018	Commercial Human Fibrin	1-Polyethylene glycol (PEG)	Monolayer TESS	In vivo	Yorkshire Pigs/Deep Partial-Thickness Burns	Porcine Adipose Tissue derived-MSCs	The combination of PEG-fibrin hydrogels and MSCs increased blood vessel size (higher expression of CD31 protein levels)
[82] / 2018	Commercial Human Fibrin	1-Polyethylene glycol (PEG)	Monolayer TESS	In vitro	-	Human Fibroblasts	In comparison with growth on collagen, fibroblasts seemed to survive and proliferate to a higher extent on fibrin
[83] / 2018	Platelet-Rich Plasma (PRP) from Human Blood Plasma	1-Alone	Wound Dressing	In vivo	Mice/Diabetic Chronic Ulcers	-	Low (2 × 10^6^ platelets/µL) and high (10 × 10^6^ platelets/µL) concentrations of PRP induced wound healing, however, the higher resulted in a slowdown of the membrane resorption that interfered with the skin healing
[84] / 2018	Commercial Human Fibrin	1-Modified Cellulose	Monolayer TESS	In vitro	-	Human Fibroblasts	The combination of fibrin mesh and cellulose supported the cell attachment and the subsequent proliferation
[85] / 2018	Commercial Human Fibrin	1-Polylactic acid (PLA)	Monolayer TESS	In vitro	-	Human Dermal Fibroblasts	Two types of fibrin nanocoating were evaluated, demonstrating that when an homogeneous mesh was formed the cell proliferation was higher
[86] / 2018	Commercial Human Fibrin and Blood Plasma from Pigs	1-Polyethylene glycol (PEG)	Wound Dressing	In vivo	Pigs/Burns	-	The PEG-fibrin hydrogel displayed less contraction than other treatments after 28 days and reduced neutrophils and macrophages in surrounding granulation tissue on day 7
[87] / 2018	Commercial Human Fibrin	1-Alginate	Monolayer TESS	In vitro	-	Human Umbilical Vein Endothelial Cells (HUVECs) or Human Fibroblasts or Keratinocytes or Human Adipose Tissue-derived MSCs	The scaffolds with low fibrinogen content (15%) showed the highest adhesiveness and survival rates for all types of cells
[88] / 2018	Human Blood Plasma	1-Agarose	Bilayer TESS	In vitro	-	Human Fibroblasts and Keratinocytes	Shrinkage was lesser in bilayer TESSs than in human and mouse skin
[89] / 2018	Commercial Human Fibrin and Platelet-Free Plasma (PFP) from Human Blood Plasma	1-Polyethylene glycol (PEG)	Wound Dressing	Ex vivo over human discarded skin	-	-	PEG-PFP hydrogel-treated wounds epithelialized faster than other treatments at days 6 to 14
[90] / 2018	Human Fibrin	1-Alone 2-Interpenetrating Polymer Networks (IPNs)	Monolayer TESS Wound Dressing	In vivo	Mice/Subcutaneous Implantation	Human Fibroblasts or Keratinocytes	Visual macroscopic observations showed that both hydrogels kept their shape and volume while the control collagen implants could barely be detected due to degradation
[91] / 2018	Human Blood Plasma	1-Tegaderm	Monolayer TESS Wound Dressing	In vivo	Rats/Excisional Wounds	Rat Fibroblasts	In the cellular group, the re-epithelialization rate, the fibroblast percentage and the collagenization in all timepoints were significantly higher
[16] / 2019	Human Blood Plasma	1-Alone	Monolayer TESS	In vitro	-	Human Fibroblasts	Remarkable improvement in the mechanical properties of the human blood plasma gels was detected when the two highest transglutaminase concentration were tested (10 and 12.5 U/g)
[92] / 2019	Platelet-Rich Plasma (PRP) from Human Blood Plasma	1-Alone 2-Acellular Dermal Matrix (ADM)	Wound Dressing	In vivo	Mice/Excisional Full-Thickness Wounds	-	The application of the ADM/PRP wound dressing significantly promoted the wound healing rate, revascularization and epithelialization
[93] / 2019	Platelet-Rich Plasma (PRP) from Human Blood Plasma	1-Collagen/Glycosaminoglycan (GAG)	Bilayer TESS	In vivo	Chick Chorioallantoic Membrane (CAM) Assay	Human Fibroblasts and Keratinocytes	Collagen-GAG-PRP scaffolds demonstrated an increased angiogenic and vascularization potential
[94] / 2019	Human Blood Plasma	1-Polyethylene glycol (PEG)	Monolayer TESS	In vivo	Rats/Full-Thickness Skin Wounds	Human Adipose Tissue-derived MSCs	The combination of PEG-plasma and MSCs showed a sooner formation of vessels and in far greater abundance
[95] / 2019	Commercial Human Fibrin	1-Alone	Trilayer TESS	In vitro	-	Human Endothelial Cells, Human Fibroblasts and Keratinocytes (and Macrophages)	Endothelial cells and dermal fibroblasts formed capillary-like structures within the dermis whereas the keratinocytes formed an epithelial cell layer
[96] / 2019	Commercial Human Fibrin	1-Alone	Trilayer TESS	In vitro	-	Human Adipose Tissue derived-MSCs, Human Fibroblasts and Keratinocytes	Protocol description
[97] / 2019	Commercial Platelet-Free Plasma (PFP) from Human Blood Plasma	1-Polyethylene glycol (PEG)	Monolayer TESS Wound Dressing	In vivo	Rats/Excisional Full-Thickness Wounds	Human Adipose Tissue derived-MSCs	Hydrogels combined with MSCs exhibited an increase in blood vessel density
[98] / 2019	Human Blood Plasma	1-Alone	Wound Dressing	In vitro	-	-	The fibrin matrix mass determined the protein retain of the serum used for culture and their release over time
[29] / 2020	Human Blood Plasma	1-Agarose 2-Hyaluronic Acid	Bilayer TESS Wound Dressing	In vivo	Mice/Excisional Surgical Wounds	Human Fibroblasts and Keratinocytes	Bilayer TESSs showed a proper clinical integration and epithelization after eight weeks
[99] / 2020	Platelet-Rich Fibrin (PRF) from Human Blood Plasma	1-Alone	Wound Dressing	In vitro	-	-	The combination of PRF with foam-based wound dressings is not recommended because of their persistent retention of growth factors
[100] / 2020	Human Blood Plasma	1-Agarose	Monolayer TESS	In vitro	-	Human Fibroblasts	Functionalization of fibrin-agarose human dermal substitutes with antibiotics was able to improve the antibacterial and biomechanical properties of the TESSs
[101] / 2020	Human Blood Plasma	1-Agarose	Bilayer TESS Monolayer TESS (Fibroblasts)	In vitro	-	Human Fibroblasts and Keratinocytes	The optical properties revealed that the bilayer TESS better resembled the optical behavior of the native human skin
[102] / 2020	Human Blood Plasma	1-Alone	Bilayer TESS	In vitro	-	Human Fibroblasts and Keratinocytes	Bioprinting protocol
[103] / 2020	Commercial Human Fibrin	1-Poly (Nisopropylacrylamideco- acrylic acid) (p(NIPAAmAA))	Trilayer TESS	In vitro	-	Human Umbilical Vein Endothelial Cells (HUVECs), 3T3 Cell Line, Human Keratinocytes (HaCaTs)	High cell viability of HUVECs, fibroblasts and keratinocytes were reported Superficial cornification of the epidermis layer as well as sprouting and splitting of the subcutaneous endothelial cells were evidenced by histology
[104] / 2020	Human Blood Plasma: Neonatal and Adult	1-Alone	Wound Dressing	In vivo	Mice/Excisional Wounds	-	Significantly smaller wound areas and greater epidermal thickness were observed when wounds were treated with neonatal fibrin compared
[105] / 2021	Commercial Human Blood Plasma	1-Catechol/Hyaluronic Acid/Alginate	Bilayer TESS Monolayer TESS (Human Fibroblasts or Keratinocytes)	In vitro	-	Human Fibroblasts and Keratinocytes	The scaffold presented high elasticity and supported the formation of a double-layered cell-laden skin like structure
[106] / 2021	Human Blood Plasma	1-Hyauronic Acid/Polyethylene glycol diacrylate (PEGDA)	Bilayer TESS	In vitro	-	Human Fibroblasts and Keratinocytes	Blood Plasma-Hyaluronic Acid-PEGDA hydrogels were up to three times thicker than the plasma controls, evidencing a reduction in contraction, while they also showed better and more homogeneous Keratin 10 expression in the supra-basal layer of the epidermis
[107] / 2021	Commercial Human Fibrin	1-Alone	Others: Injection	In vivo	Mice/Intradermal Injection on Diabetic Ulcers	-	Once the fibrin was degraded after the first week, the induced angiogenesis mostly regressed by 4 weeks, but it promoted effective arteriogenesis in the dermal layer
[108] / 2021	Human Blood Plasma	1-Alone	Wound Dressing	In vivo	Mice/Full-Thickness Wounds	-	Hydrogel significantly improved dermal repair and vascularization
[109] / 2021	Commercial Human Fibrin	1-Alginate	Wound Dressing	In vivo	Pigs/Surgical Wounds	-	The use of fibrin scaffold increased and sustained superficial blood flow and reduced contraction during early healing while showing comparable wound closure, re-epithelialization and final wound outcome to other treatments
[110] / 2021	Human Blood Plasma	1-Alone	Bilayer TESS	In vitro	-	Human Fibroblasts and Keratinocytes	A higher concentration of fibrin (2.4 mg/mL) reduced gel contraction and allowed the growth and proliferation of primary cells
[111] / 2021	Human Blood Plasma	1-Alone	Bilayer TESS	In vitro	-	Human Fibroblasts and Keratinocytes	Gels showed a height reduction of 30% during the first 24 h likely due to the intrinsic fibrin matrix contraction
[112] / 2021	Human Blood Plasma	1-Alone 2-Elastin	Bilayer TESS	In vitro	-	Human Fibroblasts and Keratinocytes	Fibroblasts proliferation was improved when lowest elastin content was applied (1 wt.%) When elastin content was 5 wt.% an increased proliferation of the keratinocytes was reported
[113] / 2021	Commercial Human Fibrin	1-Polyethylene glycol (PEG)	Bilayer TESS Others: Injection	In vivo	Mice/Subcutaneous Injection	Human Endothelial Cells and Human Fibroblasts	High concentration (250 mM) of salt (NaCl) during gel fabrication increased stability of the scaffolds and maintained cell viability
[114] / 2021	Commercial Human Fibrin	1-Alone 2-Agarose	Microspheres	In vivo	Chick Chorioallantoic Membrane (CAM) Assay	Human Umbilical Cord Blood derived-MSCs and Human Fibroblasts	Migrating fibroblasts proliferated and produced endogenous ECM, forming a dense tissue After only 4 days, perfused chimeric capillaries with human cells were present in proximal areas
[115] / 2021	Commercial Human Fibrin (Tisseel)	1-Alone	Wound Dressing	In vivo	Rabbits/Chronic Ischemic Wounds	-	The use of Tisseel combined with exosomes promoted full-thickness healing associated with collagen synthesis and restoration of the dermal architecture
[116] / 2021	Human Blood Plasma	1-Alone 2-Silica 3-Silica/Chitosan	Wound Dressing	In vitro	-	-	0.7 mg/mL chitosan-silica improved the mechanical stability of the fibrin hydrogels with low risks of cytotoxicity
[117] / 2022	Commercial Human Fibrin	1-Silk fibroin/Hyaluronic Acid	Wound Dressing	In vivo	Rabbits/Full-Thickness Burn Wounds	-	The fibrin composite scaffold promoted healing with mature epithelium coverage, dermal regeneration with angiogenesis, and deposition of remodeled ECM
[118] / 2022	Commercial Human Fibrin	1-Hyaluronic Acid/Poly(l-lactide-coglycolide-cocaprolactone) (PLGC)	Wound Dressing	In vivo	Rabbits/Full-Thickness Burn Wounds	-	Burns healed were comparable to native skin with a complete regeneration
[119] / 2022	Human Blood Plasma	1-Agarose	Bilayer TESS	In vitro	-	Human Fibroblasts and Keratinocytes	UV absorbance increased, and UV transmission decreased with culture time, and comparable results to the control were found at 21 and 28 days
[120] / 2022	Human Blood Plasma	1-Alone 2-Agarose	Monolayer TESS Wound Dressing	In vitro	-	Human Fibroblasts	The addition of agarose increased the stiffness whereas the porosity decreased Fibroblasts seeded in low plasma-agarose concentrations spread faster for 14 days
[121] / 2022	Human Blood Plasma	1-Alone	Bilayer TESS Wound Dressing	In vivo	Mice/Full-Thickness Wounds	Human Fibroblasts and Keratinocytes	A keratinized epithelium over a dermal layer was observed after 21 days (Involucrin, Keratin 10 and Vimentin expression)
[122] / 2022	Platelet-Rich Plasma (PRP) from Human Blood Plasma	1-Gelatin methacrylate (GelMA)	Wound Dressing	In vivo	Chick Chorioallantoic Membrane (CAM) Assay	-	3D printed PRP-GelMA scaffolds facilitated the controlled release of growth factors for up to 14 days, presenting superior angiogenic potential
[123] / 2022	Commercial Human Fibrin (Tisseel)	1-Alone	Monolayer TESS Others: Spray	In vitro	-	Human Keratinocytes	There was a significant increase in vitality while cultivating the cells in fibrin Sprayed cells were considerably more homogenously distributed
[124] / 2022	Human Blood Plasma and Blood Plasma from Rats	1-Alginate/Gelatin	Bilayer TESS	In vivo	Rats/Full-Thickness Wounds	Rat Fibroblasts and Keratinocytes	Blood plasma facilitated vital physiological processes including ECM synthesis, macrophage polarization, and angiogenesis
[125] / 2023	Commercial Human Fibrin	1-Collagen	Bilayer TESS	In vitro	-	Human Fibroblasts and Keratinocytes	A 3D platform containing nociceptor-innervated skin of human origin was developed and the potential to evaluate the effect of topical compounds on innervating fibers was demonstrated
[126] / 2023	Platelet-Rich Plasma (PRP) from Human Blood Plasma	1-Hyaluronic Acid	Wound Dressing	In vivo	Rats/Full Skin Defects	-	Hydrogels with PRP showed superior therapeutic effects in reducing inflammatory response, promoting collagen deposition, facilitating re-epithelialization and angiogenesis
[127] / 2023	Human Blood Plasma	1-Alone	Monolayer TESS	In vivo	Mice/Subcutaneous Implantation	Human Adipose Tissue-derived MSCs, differentiated to Adipocytes	The addition of differentiated MSCs into the graft increased the integration within the tissue
[128] / 2023	Commercial Human Fibrin	1-Alone	Monolayer TESS Wound Dressing	In vivo	Mice/Full-Thickness Skin Wounds	Human Umbilical Cord Blood-derived MSCs	The combination of fibrin and MSCs increased Keratin 10 and Keratin 14 expression and promoted wound closure, re-epithelialization and neovascularization in a better way than the wound dressing
[129] / 2023	Commercial Human Fibrin	1-Alone	Wound Dressing	In vivo	Mice/Full-Thickness Excisional Wounds	-	Fibrin hydrogel reduced the expression of pro-inflammatory cytokines and increased IL-10 levels. Moreover, keratinocyte migration was enhanced and the healing of the wounds was accelerated
[130] / 2023	Human Plasma	1-Alone 2-Graphene Oxide (GO)	Wound Dressing	In vitro	-	-	The fibrin-derived hydrogels containing GO and antibiotics showed a dose-response behavior according and allowed a sustained release of the antibiotic at a programmed rate, leading to drug delivery over a prolonged period of time. Moreover, the presence of fibrin increased cell viability of human dermal fibroblasts
[131] / 2023	Human Plasma	1-Alone 2-Hyaluronic Acid 3-Collagen	Bilayer TESS	In vitro	-	Human Fibroblasts and Keratinocytes	No significant differences regarding mechanical and biological properties among the different types of HPSS developed and well-differentiated epidermis was achieved in all cases
[132] / 2023	Human Plasma	1-Agarose	Monolayer TESS	In vitro	-	Human Wharton’s jelly-derived MSCs	The Monolayer TESSs developed, increased cell proliferation without altering cell phenotype of immunogenicity
[133] / 2024	Commercial Human Fibrin (Tisseel)	1-Alone	Sealant/Glue	In vivo	Rabbits/Full-Thickness Excisional Wounds	-	Slower healing was obtained and wound area reduction was also lesser when fibrin glue was only applied onto the wounds, compared with other biological treatments with or without cells
[134] / 2024	Platelet-Rich Plasma (PRP) from Human Blood Plasma	1-Poly Glycerol Sebacate/Polylactic acid/Platelet-Rich Plasma (PGS/PLA/PRP)	Wound Dressing	In vitro	-	-	The presence of PRP in the PGS/PLA-PRP scaffolds led not only to enhanced collagen deposition and angiogenesis but also to controlling inflammation factors involved in wound healing
[135] / 2024	Human Platelet-Rich Fibrin and Plasma (PRF and PRP)	1-Alone	Others: Injection	In vitro	-	-	Both PRP and PRF facilitated dermal cells proliferation, and migration, along with trichogenic inductivity while the impacts of PRF was more significant than PRP.
[136] / 2024	Commercial Fibrinogen from Human Plasma	1-Collagen	Bilayer TESS	In vitro	-	Human Fibroblasts and Keratinocytes	When combined with tilapia fish collagen, fibrinogen skin equivalents displayed similar morphological features to native human skin and possessed good barrier integrity
[137] / 2024	Human Commercial Fibrinogen	1-Alone	Bilayer TESS	In vivo	Mice/Surgical Wounds	Human Fibroblasts and Keratinocytes	The hydrogel structure had relatively large pore sizes that facilitated cell migration. These conditions enhanced cell viability, supported cell division and migration, and facilitated cellular rearrangement

**Table 5:** Clinical studies using human blood plasma, fibrinogen or fibrin as a biomaterial for the manufacture of dermatological treatment approaches.

Reference / Year	Plasma/Fibrinogen/Fibrin Source	Type of Clinical Study	Dermatological Treatment Approach / Cells	N (Male/Female)	Indication	* Outcomes
[181] / 1977	Allogeneic	Case Report	Wound dressing /	40 (32/8)	Surgically created periorbital skin defects	Grafts were gradually replaced by new epithelial tissue growing in from the periphery of the wound edge
[182] / 2004	Allogeneic (Combined with Integra^®^)	Case Report	As a glue combined with Integra^®^ for temporary wound dressing before skin transplantation (Evaluation of the effect of negative pressure therapy) /	12 (7/5)	Acute and chronic wounds	The mean period from Integra coverage to skin transplantation was 24 ± 3 days in the conventional treatment group but only 10 ± 1 days in the fibrin/negative-pressure therapy group
[183] / 2004	Allogeneic	Case Report	Bilayer TESSs / Autologous fibroblasts and keratinocytes	2 (2/0)	Burns	Epidermal regeneration analyzed 2 months after grafting was complete (100% of the grafted area)
[184] / 2004	Autologous	Case Report	Monolayer TESSs / Allogeneic ^a^ hUCB-MSCs (^b^ HLA compatible)	2 (1/1)	Trauma and radiation injuries	The level of improvement, scored arbitrarily from 0 to 4, was 3 and 4
[185] / 2006	Allogeneic	Case Series	Bilayer TESSs / Autologous fibroblasts and keratinocytes	20	13—Burns 5—Giant nevus 1—Graft versus host disease 1—Neurofibromatosis	The engineered skin took in all patients. The epithelization obtained was permanent in all cases
[196] / 2011	Allogeneic	Multicenter retrospective observational cohort study	Bilayer TESSs / Autologous fibroblasts and keratinocytes	25 (23/2)	Burns	Characteristic scarring of mesh interstices was avoided. Epithelialization was observed
[193] / 2015	Autologous (Combined with gelatin or hydrocolloid dressings)	Open-label, Non-Randomized, Controlled Clinical Trial	Wound dressing / ---	Estimation:30	Difficult to heal chronic skin ulcers	Protocol description
[186] / 2015	Allogeneic	Case Series	Bilayer TESSs / Autologous fibroblasts and keratinocytes	8 (3/5)	Hypertrophic scars due to full-thickness burns	After 8 weeks, re-epithelialization and reduction in hypertrophic scars were achieved in 2 patients Decreased pain rate
[187] / 2016	Autologous	Case Report	As temporary wound dressing before skin graft / ---	1 (1/0)	Refractory skin ulcer	Process of re-epithelialization was completed after 4 months
[188] / 2016	Allogeneic	Case Report	Bilayer TESSs / Autologous fibroblasts and keratinocytes	2 pediatric patients	Burns	Appearance of the skin did not differ significantly from the areas treated with autografts
[194] NCT03113747 / 2017	No tissue source indicated	Randomized Clinical Trial Parallel Assignment (Open Label) Phases 1 and 2	Monolayer TESSs / Allogeneic hAT-MSCs (Followed by autografts)	Estimation: 20	Burns	No results posted yet
[189] / 2019	Allogeneic (Combined with agarose)	Case Series	Bilayer TESSs / Autologous fibroblasts and keratinocytes	9 adult patients (7/2) 3 pediatric patients (1/2)	Burns	No results posted yet
[195] IRCT2015110224834N1 / 2020	Allogeneic (Combined with human collagen)	Clinical Trial	Trilayer TESSs and Bilayer TESSs / Autologous hAT-MSCs, fibroblasts and keratinocytes	5 Trilayers (4/1) 5 Bilayers (4/1)	Diabetic foot ulcers	Increased skin thickness and density in the vascular beds of the hypodermis of trilayer treated patients
[190] / 2020	Autologous (Combined with allogeneic human amnion)	Case Report	Wound Dressing /	1 (1/0)	Toxic epidermal necrolysis	Significant acceleration of wound healing (6 days)
[197] / 2020	Autologous	Prospective clinical analysis (Pre–post pilot study)	Monolayer TESSs / Autologous hAT-MSCs	6 (3/3)	Chronic diabetic ulcers	There was granulation tissue formation starting from 7 days after topical application. After 90 days, a healed and re-epithelialized tissue was observed
[191] / 2021	Autologous	Case Series	Wound Dressing /	1 (1/0)	Non-healing wounds	After 7 days no maceration and oozing were observed After 3 injections wound was completely healed
[192] / 2021	Autologous	Case Report	Wound Dressing /	1 (0/1)	Reconstruction of exposed skull in a complex craniovertebral polytrauma	Application of plasma facilitated wound regeneration
* Expression of measures: mean +/− standard deviation (range). No treatment-related adverse events were reported.						
^a^ hUCB-MSCs: human umbilical cord blood mesenchymal stem cells. ^b^ HLA: human leukocyte antigen.						

## Data Availability

No new data were created or analyzed in this study. Data sharing is not applicable to this article.

## Supplementary Materials

The following supporting information can be downloaded at: https://www.mdpi.com/article/10.3390/jfb16030079/s1, Supplementary Table S1. Dermatological treatment approaches investigated using blood plasma, fibrinogen or fibrin as a biomaterial: type and number of studies reviewed; Supplementary Table S2. Biomaterials combined with blood plasma, fibrinogen or fibrin for the manufacture of dermatological treatment approaches; type and number of studies reviewed.

## Abbreviations

The following abbreviations are used in this manuscript:

3D	Three dimensional
ADM	Acellular dermal matrix
AMP	Antimicrobial peptides
BiOCl	Bismuth oxychloride
Ca2+	Calcium
CAM	Chorioallantoic membrane
dsECM	Decellularized human skin-derived extracellular matrix
ECM	Extracellular matrix
ELNs	Exosome-like nanoparticles
FGF	Fibroblast growth factor
GelMA	Gelatin methacrylate
GO	Graphene oxide
GP	β-glycerophosphate
HA	Hyaluronic acid
HAMA	Methacrylated hyaluronic acid
hAT-MSCs	Human adipose tissue-derived MSCs
hBM-MSCs	Human bone marrow-derived MSCs
HEMA	2-hydroxyethyl methacrylate
HPMA	2-hydroxypropyl methacrylate
HPC	Hydroxy propyl cellulose
hPRP	Human platelet-rich plasma
IPNs	Interpenetrating polymer networks
MSCs	Mesenchymal stem cells
NB	Novobiocin sodium salt
p(NIPAAmAA)	Poly (N-isopropylacrylamide-co-acrylic acid)
PAA	Polyacrylic acid
Pec	Pectin
PEG	Polyethylene glycol
PEGDA	Polyethylene glycol diacrylate
PFP	Platelet-free plasma
PGA	Polyglycolic acid
PGS	Poly glycerol sebacate
PLA	Polylactic acid
PLGC	Poly(l-lactide-co-glycolide-co-caprolactone)
PLGA	Polylactic-co-glycolic acid
SCH	Sildenafil citrate hydrogel
TE	Tissue engineering
TESS	Tissue-engineered skin substitutes
VEGF	Vascular endothelial growth factor
