# Supplementary material for: Blood Plasma, Fibrinogen or Fibrin Biomaterial for the Manufacturing of Skin Tissue-Engineered Products and Other Dermatological Treatments: A Systematic Review
Source: J Funct Biomater. 2025 Feb 22;16(3):79. doi: 10.3390/jfb16030079 (PMC11942893; doi:10.3390/jfb16030079)
Supplement: Supplementary file 1 [file jfb-16-00079-s001.zip › jfb-3461451-supplementary.pdf]

Supplementary Table S1. Dermatological treatment approaches investigated using blood plasma, fibrinogen or fibrin as a biomaterial: type and number of studies reviewed.

**Dermatological treatment approach**	***In vitro* studies / References**	***In vivo* studies* / References**	**Total number of studies**	****Studies evaluating two or more approach**
**Wound dressing**	8 [36,43,98,99,116,120,130,134]	40 [29,57,59,65,71,80,83,86,89–92,97,104,108,109,115,117,118,121,122,126,128,129,138,139,143,149,153,156,158–160,163,166–168,174–176]	48	15
**Monolayer TESSs**	30 [16,37,44,45,50,54,64,72,74,76,82,84,85,87,90,100,101,105,120,123,132,140,144–146,148,152,154,161,169]	18 [35,38,46,51,60,66,70,81,91,94,97,127,128,147,150,156,170,173]	48	
**Bilayer TESSs**	24 [40,42,45,47,49,52,55,63,72,75,77,88,93,101,102,105,106,110–112,119,125,131,136]	15 [29,33,35,39,41,56,69,113,121,124,137,155,164,170,171]	39	
**Trilayer TESSs**	7 [53,61,67,68,95,96,103]	2 [141,142]	9	
**Microspheres**	1 [79]	1 [114]	2	
**Sealant / Glue**	1 [172]	6 [32,34,48,62,133,162]	7	
**Others**	3 [73,123,135]	6 [78,113,151,157,163,165]	9	
**TOTAL**	74	89	162	
**TOTAL NUMBER OF INDIVIDUAL STUDIES REVIEWED**			162 -15 = 147	

***Ex vivo* studies and chorioallantoic membrane (CAM) assays are considered as *in vivo* studies**
****This number of studies must be subtracted to the final total number of studies to know the number of individual studies reviewed**

Supplementary Table S2. Biomaterials combined with blood plasma, fibrinogen or fibrin for the manufacture of dermatological treatment approaches; type and number of studies reviewed.

**Biomaterial's Combination**	***In vitro* studies / References**	***In vivo* studies* / References**	**Total number of studies**	****Studies evaluating two or more biomaterial's compositions**
**Blood plasma/fibrinogen/fibrin Alone**	35 [16,36,40,42–45,50,55,61,64,67,68,72,75,76,79,95,96,98,99,102,110–112,116,120,123,130,131,135,140,144,146,169]	49 [32–35,38,39,41,46,48,51,57,62,65,66,69,78,80,83,90,92,104,107,108,114,115,121,127–129,133,137,139,141–143,150,151,153,157,159,160,163–165,167,168,170,171,175]	84	29
**+Agarose**	8 [75,77,88,100,101,119,120,132]	4 [29,56,58,114]	12	
**+Polyethylene glycol (PEG)**	2 [82,140]	8 [70,81,86,89,94,97,113,149]	10	
**+Collagen**	7 [37,43,52,125,131,136,146]	2 [71,138]	9	
**+Polylactic acid / Polylactic-co-glycolic acid / Polyglycolic acid (PLA / PLGA / PGA)**	3 [74,85,161]	3 [65,162,164]	6	
**+Hyaluronic acid (HA)**	2 [63,131]	4 [29,126,139,147]	6	
**+Gelatin**	2 [73,172]	2 [78,174]	4	
**+Alginate**	2 [87,145]	1 [109]	3	
**+Collagen/PEG**	2 [49,53]	1 [60]	3	
**+Gelatin methacrylate / Methacrylated Hyaluronic Acid (GelMA / HAMA)**	0	3 [155,156,173]	3	
**+Integra®**	1 [54]	1 [38]	2	
**+Graphene Oxide (GO)**	1 [130]	0	1	
**+Silica**	1 [116]	0	1	
**+Silica / Chitosan**	1 [116]	0	1	
**+Alginate / Polymerized polydimethylsiloxane**	1 [145]	0	1	
**+Poly (N-isopropylacrylamide-co-acrylic acid) (p(NIPAAmAA))**	1 [103]	0	1	
**+Collagen / Chitosan**	1 [47]	0	1	

**+Collagen / Alginate**	1<br>[152]	0	1	
**+Collagen / Hyaluronic Acid / PEG (4S-StarPEG)**	1<br>[154]	0	1	
**+Modified Cellulose**	1<br>[84]	0	1	
**+Decellularized human skin-derived extracellular matrix (dsECM)**	1<br>[148]	0	1	
**+Catechol / HA / Alginate**	1<br>[105]	0	1	
**+Elastin**	1<br>[112]	0	1	
**+HA / Polyethylene glycol diacrylate (PEGDA)**	1<br>[106]	0	1	
**+Poly glycerol sebacate / Poly Lactide Acid (PGS / PLA)**	1<br>[134]	0	1	
**+Chitosan**	0	1<br>[158]	1	
**+Fibronectin**	0	1<br>[139]	1	
**+Poly(ether)urethane-polydimethylsiloxane**	0	1<br>[59]	1	
**+Tegaderm**	0	1<br>[91]	1	
**+Acellular Dermal Matrix (ADM)**	0	1<br>[92]	1	
**+Sildenafil citrate hydrogel (SCH)**	0	1<br>[160]	1	
**+HA / Poly(l-lactide-co-glycolide-co-caprolactone) (PLGC)**	0	1<br>[118]	1	
**+Silk fibroin / HA**	0	1<br>[117]	1	
**+Polyvinyl alcohol**	0	1<br>[153]	1	
**+Gelatin / 2-Hydroxyethyl methacrylate (HEMA)**	0	1<br>[174]	1	
**+Gelatin / 2-Hydroxypropyl methacrylate (HPMA)**	0	1<br>[174]	1	
**+Laminin-Heparin binding domains**	0	1<br>[80]	1	
**+Alginate / Gelatin**	0	1<br>[124]	1	

**+Gelatin methacrylate (GelMA)**	0	1<br>[122]	1	
**+Collagen / Glycosaminoglycan (GAG)**	0	1<br>[93]	1	
**+Rose-derived exosome-like nanoparticles (ELNs) / Antimicrobial peptides (AMP) / Novobiocin sodium salt (NB)**	0	1<br>[175]	1	
**+Bismuth Oxychloride (BiOCl)**	0	1<br>[157]	1	
**+Pectin / Polyacrylic acid (Pec / PAA)**	0	1<br>[166]	1	
**+Chitosan / β-glycerophosphate (GP) / Hydroxy Propyl Cellulose (HPC) / Graphene Oxide (GO)**	0	1<br>[176]	1	
**+Interpenetrating Polymer Networks (IPNs)**	0	1<br>[90]	1	
**TOTAL**	78	98	176	
**TOTAL NUMBER OF INDIVIDUAL STUDIES REVIEWED**				176 – 29 = 147

***Ex vivo* studies and chorioallantoic membrane (CAM) assays are considered as *in vivo* studies**
****This number of studies must be subtracted to the final total number of studies to know the number of individual studies reviewed**
